# Pericyte-to-endothelial cell signaling via vitronectin-integrin regulates blood-CNS barrier

**DOI:** 10.1016/j.neuron.2022.02.017

**Published:** 2022-03-15

**Authors:** Swathi Ayloo, Christopher Gallego Lazo, Shenghuan Sun, Wei Zhang, Bianxiao Cui, Chenghua Gu

**Affiliations:** 1 Howard Hughes Medical Institute, Department of Neurobiology, Harvard Medical School, Boston, MA 02115, USA; 2 Department of Chemistry, Stanford University, Stanford, California 94305, USA; 3 Current address: Sanofi-GMU, 225 2^nd^ Avenue, Waltham, MA 02451; 4 Current address: Biomedical Sciences Graduate Program, UCSF, San Francisco, CA 94143, USA

## Abstract

Endothelial cells of blood vessels of the central nervous system (CNS) constitute blood-CNS barriers. Barrier properties are not intrinsic to these cells; rather they are induced and maintained by CNS microenvironment. Notably, the abluminal surface of CNS capillaries are ensheathed by pericytes and astrocytes. However, extrinsic factors from these perivascular cells that regulate barrier integrity are largely unknown. Here, we establish vitronectin, an extracellular-matrix protein secreted by CNS pericytes, as a regulator of blood-CNS barrier function via interactions with its integrin receptor, α5 in endothelial cells. Genetic ablation of vitronectin or mutating vitronectin to prevent integrin binding as well as endothelial-specific deletion of integrin α5 causes barrier leakage in mice. Furthermore, vitronectin-integrin α5 signaling maintains barrier integrity by actively inhibiting transcytosis in endothelial cells. These results demonstrate that signaling from perivascular cells to endothelial cells via ligand-receptor interactions is a key mechanism to regulate barrier permeability.

## eTOC blurb

Ayloo et al. reveal a mechanism of how blood-CNS barrier is actively maintained by brain parenchyma cells. They discovered a ligand-receptor signaling between pericyte-secreted vitronectin and its endothelial cell receptor integrin α5 is critical to suppress transcytosis to ensure barrier integrity in mice.

## Introduction

The CNS requires an optimal and tightly regulated microenvironment for efficient synaptic transmission. This is achieved by blood-CNS barriers that regulate substance flux to maintain tissue homeostasis. Two such barriers are the blood-brain barrier (BBB) and blood-retina barrier (BRB) that are physiologically similar barriers separating the blood from brain and retina, respectively. The restrictive permeability of CNS endothelial cells that constitute these barriers is a result of specialized tight junctions and low rates of transcytosis, which limit substance exchange between blood and the CNS tissue (Andreone et al., 2017; Ben-Zvi et al., 2014; Chow and Gu, 2017; Langen et al., 2019; Reese and Karnovsky, 1967; Zhao et al., 2015).

Barrier properties are not intrinsic to CNS endothelial cells; they require active induction and maintenance from brain parenchyma cells (Armulik et al., 2010; Daneman et al., 2010; Heithoff et al., 2021; Stewart and Wiley, 1981). For example, Wnt ligands released by glia and neurons act on CNS endothelial cells to induce and maintain barrier properties (Daneman et al., 2009; Liebner et al., 2008; Stenman et al., 2008; Wang et al., 2012, 2019b). In contrast to glia and neurons, pericytes are directly in contact with capillary endothelial cells. Pericytes ensheathe capillary endothelial cells and share the same basement membrane with them allowing for elaborate cell-cell signaling between these two cells. Intriguingly, the brain and retina have the highest pericyte to endothelial cell ratio compared to that in other tissues (Frank et al., 1987; Shepro and Morel, 1993). Indeed, mice with decreased pericyte coverage around endothelial cells exhibit leaky blood-CNS barriers (Armulik et al., 2010; Bell et al., 2010; Daneman et al., 2010; Park et al., 2017). However, how pericytes signal to endothelial cells to maintain barrier integrity is unknown.

Here, we identify vitronectin, a pericyte-secreted extracellular matrix (ECM) protein as an important regulator of barrier integrity. We establish that vitronectin regulates barrier function via binding to its integrin receptors on endothelial cells. Specifically, vitronectin is enriched in CNS pericytes, and mice lacking vitronectin as well as vitronectin mutant mice (*Vtn*^*RGE*^) that cannot bind integrin receptors exhibit barrier leakage. Moreover, we found that the RGD-ligand binding integrins, α5 and αv are expressed in CNS endothelial cells, and endothelial cell-specific acute deletion of α5 but not αv results in leaky barrier. We further demonstrate that barrier leakage observed in vitronectin mutant mice is due to increased transcytosis in CNS endothelial cells, but not due to deficits in tight junctions; and activation of integrin α5 by vitronectin inhibits endocytosis in CNS endothelial cells. Together, our results indicate that ligand-receptor interactions between pericyte-derived vitronectin and its integrin receptor expressed in CNS endothelial cells are critical for barrier integrity and may provide novel therapeutic opportunities for CNS drug delivery.

## Results

### Vitronectin is enriched in CNS pericytes compared to pericytes of peripheral tissues and its expression coincides with functional barrier formation

In order to identify pericyte candidate genes important for barrier function we took advantage of the retina as a model system. In the retinas of mice, CNS vessels invade the optic nerve head at postnatal day (P1) and expand radially from the center toward the periphery. As vessels grow from the proximal to distal ends of the retina, proximal vessels gain barrier properties and have a functional BRB, while the newly formed distal vessels have a leaky BRB (Chow and Gu, 2017). By examination of an existing transcriptome comparing the proximal and distal retinal vessels that contain a mixture of endothelial cells and pericytes (Strasser et al., 2010) and a brain pericyte transcriptomic database (He et al., 2016), we identified CNS pericyte genes that correlated with functional blood-retinal barrier. Of these candidate pericyte genes, we focused specifically on secreted and trans-membrane proteins as these are likely involved in ligand-receptor interactions. These analyses identified vitronectin, an extracellular matrix protein.

**Figure 1.**
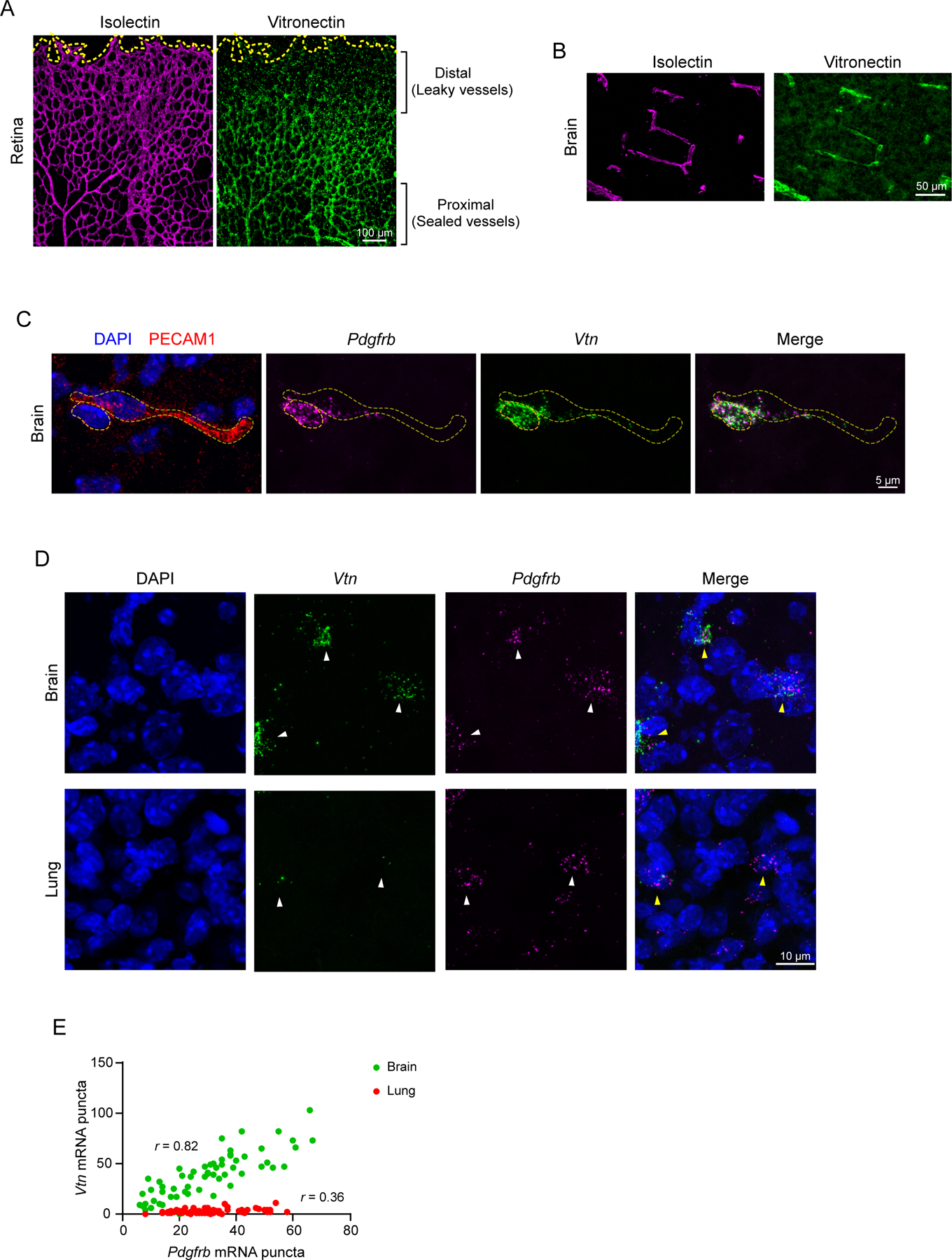
Vitronectin expression coincides with functional barrier formation and is enriched in CNS pericytes compared to peripheral tissue pericytes (A, B) Immunostaining of vitronectin (green) and blood vessels (isolectin, magenta) in retina (A) and (B) brain of P7 mouse. (C) In situ hybridization for *Vtn* (green), *Pdgfrb* (pericyte gene, magenta) and immunostaining for PECAM1 (vessel marker, red) with DAPI (nuclei, blue) in P7 brain. Dashed lines indicate pericyte nucleus and vessel outline. (D) In situ hybridization for *Vtn* and *Pdgfrb* in brain and lung of P7 mouse. (E) Scatter plot between *Pdgfrb* and *Vtn* in brain (green) and lung (red). Each dot represents an individual cell, n=60 and 62 cells respectively from N=3 animals. Pearson correlation coefficient *r* = 0.82 for brain and *r* = 0.36 for lung.

To validate our gene expression analyses, we first examined vitronectin protein localization in the CNS. Consistent with enriched *Vtn* transcript in proximal compared to distal vessels of the developing retina (Strasser et al., 2010), we also observed vitronectin protein highly expressed in proximal vessels with sealed BRB, and was not detectable in the distal, leaky vessels of the retina at P7 (Figures 1A, S1A). Similarly, vitronectin protein was also detected along capillaries of the brain (Figure 1B). As vitronectin is a secreted protein, to determine the cells producing *Vtn*, we used in situ hybridization to examine *Vtn* mRNA in the brain. *Vtn* mRNA is specifically expressed in *Pdgfrb* expressing pericytes adjacent to capillary endothelial cells (Figure 1C). At P7, *Vtn* is expressed in >97% (115 out of 118 cells across 3 mice) of the *Pdgfrb+* pericytes abutting capillaries and was present throughout the brain (Figures 1C, S1B). In contrast to high *Vtn* mRNA in CNS pericytes, we observed little to no expression in pericytes of peripheral tissues, such as the lung (Figures 1D, 1E). Our data are consistent with recent single-cell RNA sequencing studies revealing abundant *Vtn* expression in P14 retinal pericytes (Macosko et al., 2015; La Manno et al., 2021; Vanlandewijck et al., 2018). This is also consistent with single cell-RNA sequencing studies in adult brains with *Vtn* expression in pericytes but little to no expression in non-vascular CNS cells such as neurons and astrocytes (La Manno et al., 2021; Vanlandewijck et al., 2018).

### Pericyte-secreted vitronectin is required for blood-CNS barrier integrity

**Figure 2.**
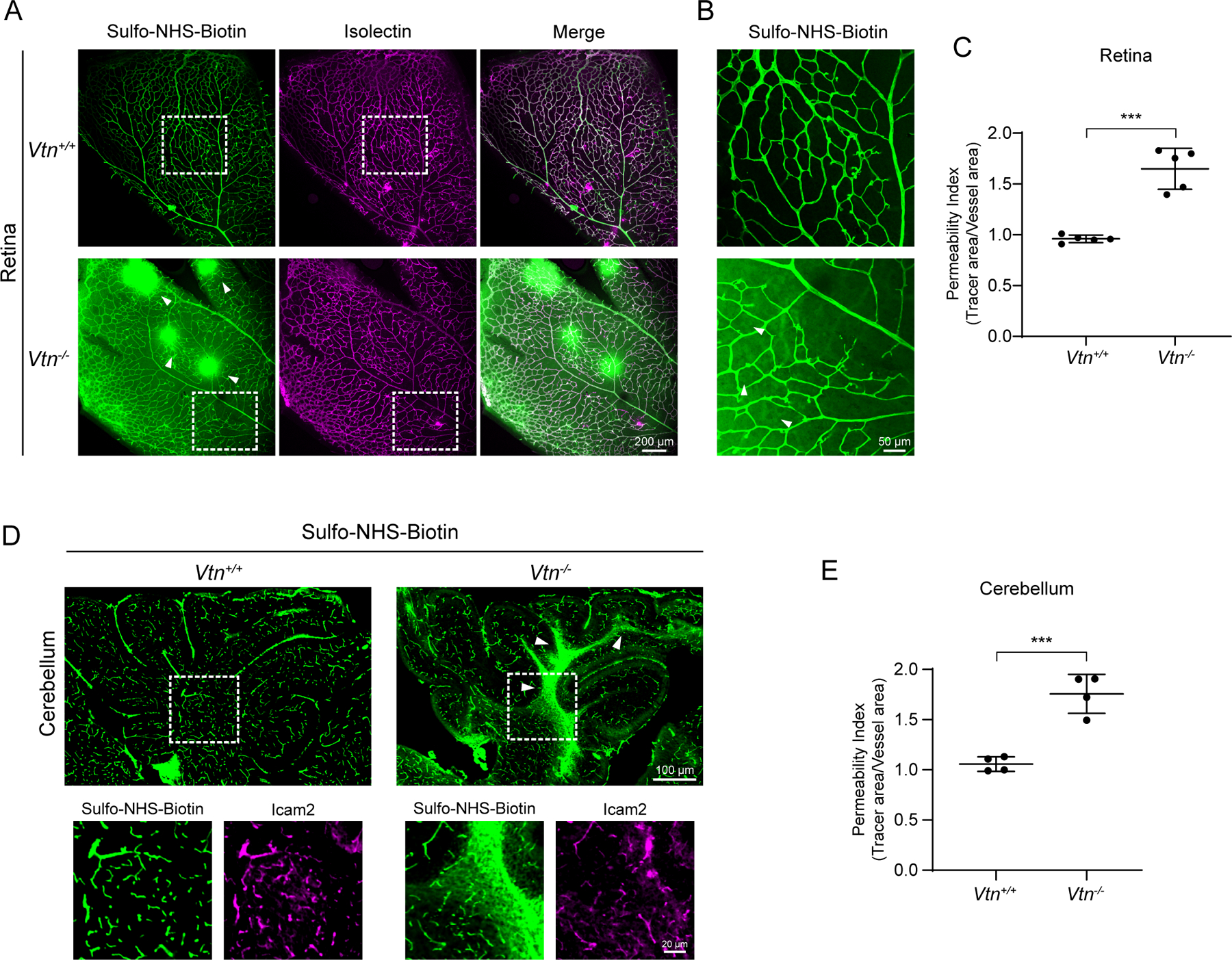
Vitronectin is essential for blood-CNS barrier integrity (A) Sulfo-NHS-Biotin (tracer, green) leaks out of blood vessels (isolectin, magenta) in retinas of P10 *Vtn*^−/−^ mice. Arrowheads show tracer hotspots in *Vtn*^−/−^ mice, white boxes correspond to higher magnification images in (B). (B) Sulfo-NHS-Biotin leaked out in *Vtn*^−/−^ mice taken up by neuronal cell bodies (arrowheads). (C) Quantification of vessel permeability in retinas of wildtype and *Vtn*^−/−^ mice. n = 5 animals per genotype. Mean ± S.D.; ***p < 0.001; Student’s t-test. (D) Leakage of Sulfo-NHS-Biotin (green) from blood vessels (Icam2, magenta) in the cerebellum of P10 *Vtn*^−/−^ mice. White boxes correspond to higher magnification panels shown. (E) Quantification of vessel permeability in retinas of wildtype and *Vtn*^−/−^ mice. n = 4 animals per genotype. Mean ± S.D.; ***p < 0.001; Student’s t-test.

To determine if vitronectin is essential for barrier function, we performed a tracer leakage assay in P10 *Vtn*^−/−^ mice when BRB and BBB are fully functional (Andreone et al., 2017; Chow and Gu, 2017). The injected tracer was completely confined to vessels in control mice, whereas tracer leaked out of vessels in the retinas of *Vtn*^−/−^ mice. Numerous leaky hotspots of the tracer were apparent in the parenchyma (Figure 2A) and in neuronal cell bodies (Figure 2B) in the retinas of these mice (Figure 2C). This leakage was not limited to just small tracers like Sulfo-NHS-Biotin (0.5 kDa) as we observed similar leakage with higher molecular weight tracers such as 10 kDa Dextran (Figure S2A, S2B). Furthermore, this leakage persisted through adulthood in these mice (Figure S2C). Similar to the retina, we also observed BBB leakage in the cerebellum (Figures 2D, 2E). Thus, these findings demonstrate an important role for vitronectin in blood-CNS barrier function.

**Figure 3.**
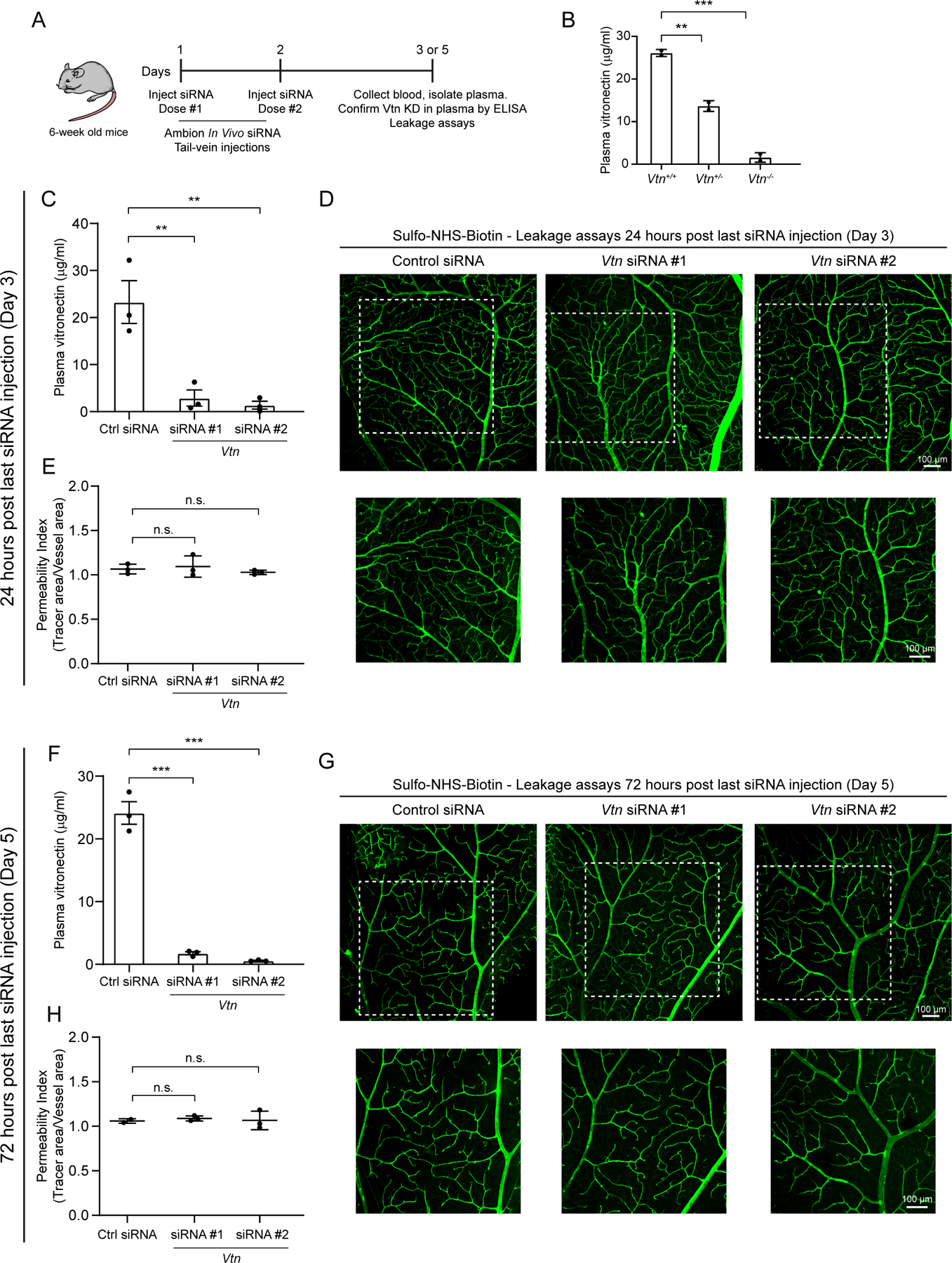
Vitronectin in plasma is not required for blood-CNS barrier function (A) Illustration of experimental paradigm to knock-down plasma vitronectin specifically with intravenous injections of siRNAs followed by vitronectin protein level measurement in plasma and evaluation of barrier integrity by leakage assays. (B) Validation of ELISA kit to measure vitronectin protein levels in plasma of wildtype, heterozygotes and vitronectin null mice. n = 2 animals per genotype. Mean ± S.D.; **p < 0.01, ***p < 0.001; one-way ANOVA with Tukey’s post hoc test. (C, F) Vitronectin levels measured by ELISA in plasma of mice injected with control (Ctrl) siRNA or two independent siRNAs targeting vitronectin. 24 hours post last siRNA injection (C) and 72 hours post last siRNA injection (F). n = 3 mice per siRNA in each case. Mean ± S.D.; **p < 0.01, ***p < 0.001; one-way ANOVA with Tukey’s post hoc test. At 24 hour time point, siRNA #1 yields 87.58 ± 1.71% and siRNA #2 yields 94.06 ± 0.83% knockdown of plasma vitronectin. At 72 hour timepoint, siRNA #1 yields 92.73 ± 0.25% and siRNA #2 yields 98.56 ± 0.06% knockdown of plasma vitronectin. (D, E) Sulfo-NHS-Biotin confined to vessels (D) in retinas of mice injected with siRNA targeting vitronectin, 24 hours post last siRNA injection. Corresponding quantification of vessel permeability (E). White boxes correspond to panel of higher magnification images. n = 3 mice per siRNA. Mean ± S.D.; n.s. not significant, p > 0.05; one-way ANOVA with Tukey’s post hoc test. (G, H) Sulfo-NHS-Biotin confined to vessels (G) in retinas of mice injected with siRNA targeting vitronectin, 72 hours post last siRNA injection. Corresponding quantification of vessel permeability (H). White boxes correspond to panel of higher magnification images. n = 3 mice per siRNA. Mean ± S.D.; n.s. not significant, p > 0.05; one-way ANOVA with Tukey’s post hoc test.

In addition to being an ECM protein, vitronectin is also an abundant protein circulating in the plasma which has been shown to mediate the complement pathway and play a role in tissue repair and wound healing (Leavesley et al., 2013; Preissner and Reuning, 2011). To fully establish that the barrier deficits we observe in *Vtn*^−/−^ mice are indeed due to the lack of pericyte-secreted vitronectin and not due to the lack of circulating vitronectin, we took advantage of the fact that vitronectin in plasma is synthesized and secreted by liver hepatocytes and intravenous injections of siRNAs largely target the liver tissue with little to no distribution to other tissues (Song et al., 2003; Zender et al., 2003). To specifically knockdown the circulating vitronectin, we intravenously injected siRNAs targeting vitronectin or control siRNAs into 6-week old wildtype mice on two consecutive days (Figure 3A) based on previously established studies for efficient liver targeting (Song et al., 2003; Wrobel et al., 2021). Knockdown of vitronectin in plasma was evaluated by ELISA at day 3 or 5, i.e. 24 hours or 72 hours post the last dose of siRNA injections (Figure 3A). ELISA kit was validated by measuring levels of plasma vitronectin in wild-type, *Vtn*^+/−^, and *Vtn*^−/−^ mice (Figure 3B). We performed these experiments with two independent siRNAs at both the timepoints. We observed as high as 98.56 ± 0.06% knockdown of vitronectin in plasma isolated from mice injected with vitronectin targeting siRNAs (Figures 3C, 3F). Importantly, at both these timepoints, we detected no leakage in mice injected with either siRNA targeting vitronectin or control siRNA (Figures 3D, 3E, 3G, 3H), demonstrating that plasma vitronectin is likely dispensable for barrier function. Thus, the combination of leaky barriers in *Vtn*^−/−^ mice and intact barriers in mice specifically lacking plasma vitronectin demonstrates an important role for pericyte-secreted vitronectin in blood-CNS barrier function.

### Vitronectin regulates blood-CNS barrier function by suppressing transcytosis in CNS endothelial cells

**Figure 4.**
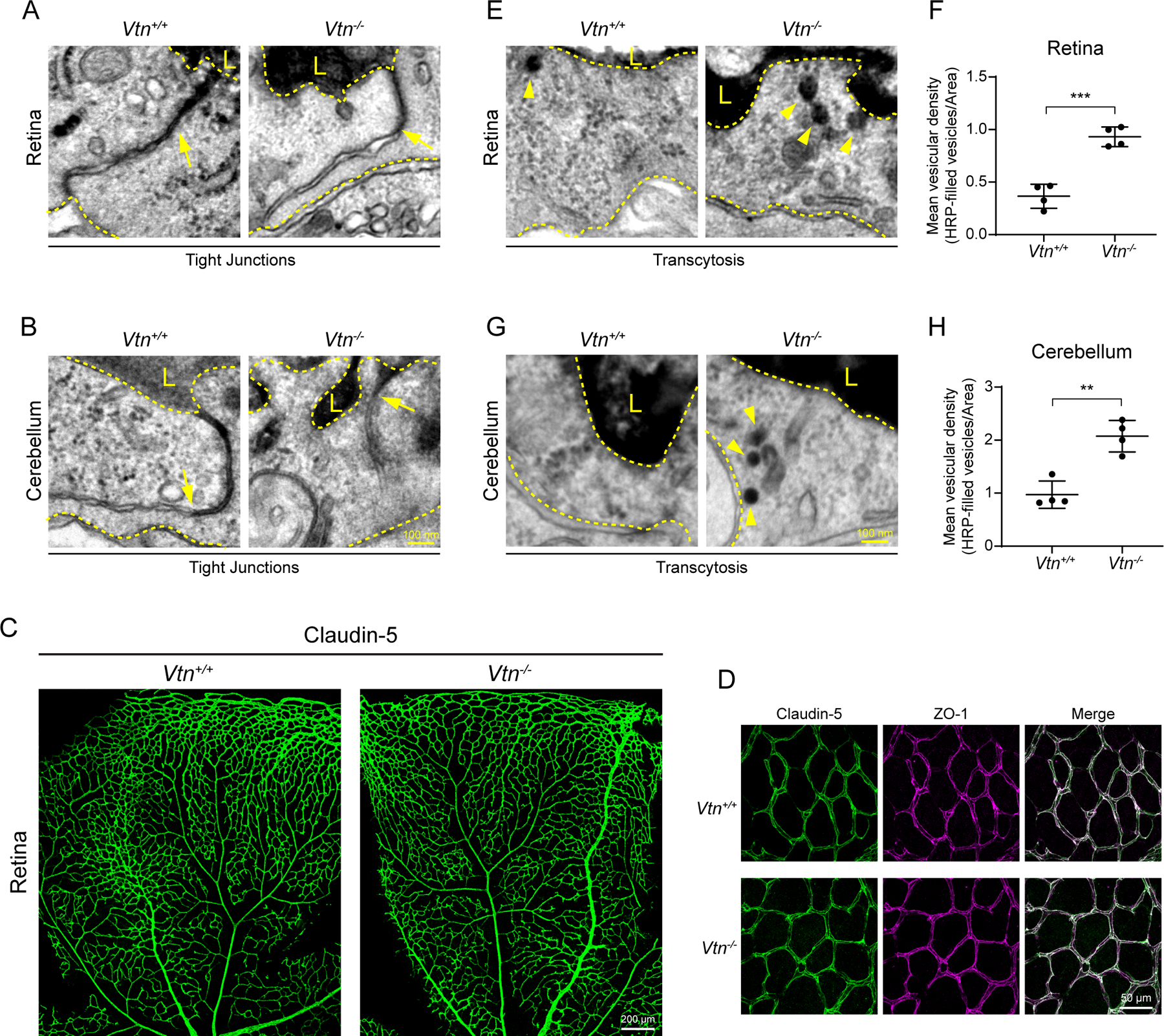
Vitronectin regulates blood-CNS barrier function by suppressing transcytosis in CNS endothelial cells (A, B) EM images of HRP halting at tight junctions (arrows) in both retinas (A) and cerebellum (B) of wildtype and *Vtn*^−/−^ mice. Luminal (L) and abluminal sides indicated by dashed yellow lines. (C) Claudin-5 immunostaining in P10 retinas of wildtype and *Vtn*^−/−^ mice. (D) Higher magnification images of Claudin-5 (green) and ZO-1 (magenta) in P10 retinas to show expression and localization of tight junction proteins at cell-cell junctions. (E, G) EM images showing HRP-filled vesicles (arrowheads) in endothelial cells of retinas (E) and cerebellum (G) of wildtype and *Vtn*^−/−^ mice. (F, H) Quantification of tracer-filled vesicles in endothelial cells of retinas (F) and cerebellum (H). n = 4 animals per genotype, 15–20 vessels per animal. Mean ± S.D.; ***p < 0.001, **p < 0.01; Student’s t-test.

To determine the subcellular basis in endothelial cells for the underlying leakage we observed in *Vtn*^−/−^ mice, we injected horse-radish peroxidase (HRP) intravenously and performed EM analysis in retina and cerebellum. In both retina and cerebellum of *Vtn*^−/−^ mice and wildtype littermates, we observed HRP halting at tight junction “kissing points” between endothelial cells of capillaries (Figure 4A, 4B), indicating functional tight junctions. Consistent with this, we observed no changes in Claudin-5 protein expression in the retinas of *Vtn*^−/−^ mice (Figure 4C). Closer examination of Claudin-5 and ZO-1 revealed that the localization of these proteins to cell-cell junctions is also unaltered (Figure 4D). In contrast, both retinal and cerebellum endothelial cells of *Vtn*^−/−^ mice exhibited significantly increased HRP-filled vesicles (Figures 4E, 4G). Retinal endothelial cells had a 3-fold increase in vesicles compared to wildtype mice (Figure 4F) and cerebellar endothelial cells had 2-fold increase (Figure 4H), revealing that transcytosis is upregulated in these mice. Interestingly, in the cerebellum of these mice, we also observed HRP-filled vesicles in adjacent pericytes (Figure S3A) and about 25% of capillaries had their basement membrane completely filled with HRP (Figures S3B, S3C). This result indicates that tracer-filled vesicles are indeed transcytosed across endothelial cells (red arrowheads in example 2 of Figure S3A). These data reveal that barrier leakage in *Vtn*^−/−^ mice is due to upregulated transcytosis. Together, these observations demonstrate that pericyte-derived vitronectin regulates barrier function by specifically inhibiting transcytosis in CNS endothelial cells.

### Vitronectin is not required for normal vessel patterning or pericyte coverage

**Figure 5.**
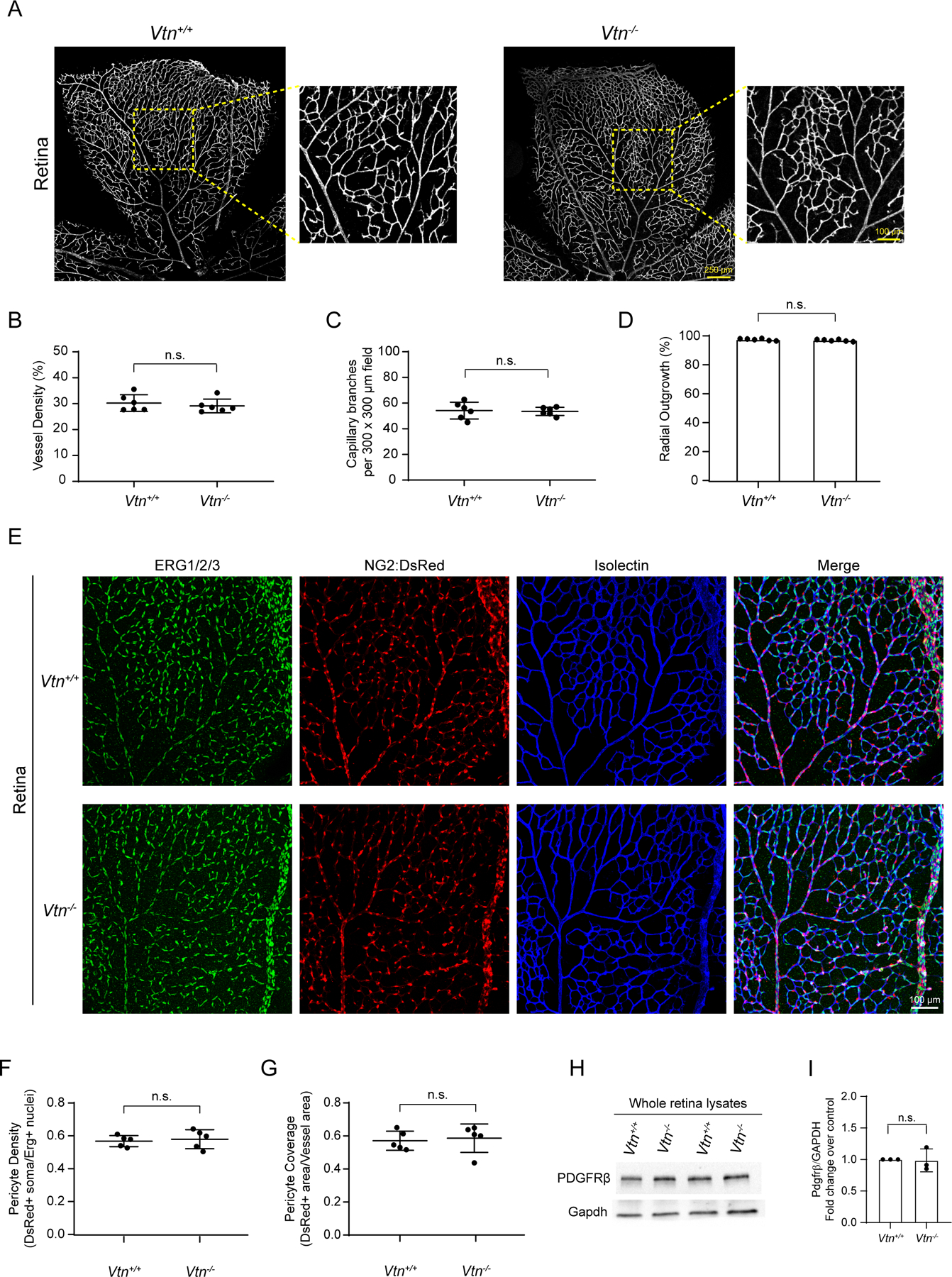
Vitronectin is not required for normal vessel patterning or pericyte coverage (A) Tilescan images showing retinal vasculature of P10 retinas in wildtype and *Vtn*^−/−^ mice. (B-D) Quantification of vessel density (B), capillary branching (C) and radial outgrowth (D) in P10 retinas. n = 6 animals per genotype. Mean ± S.D.; n.s. not significant, p > 0.05; Student’s t-test. (E) P10 retinas of NG2:DsRed+ wildtype and *Vtn*^−/−^ mice immunostained for ERG1/2/3 (endothelial nuclei, green) and vessels (isolectin, blue). (F, G) Quantification of pericyte coverage (F) and pericyte density (G) in retinas of wildtype and *Vtn*^−/−^ mice. n = 5 animals per genotype. Mean ± S.D.; n.s. not significant, p > 0.05; Student’s t-test. (H, I) Representative western blots (H) and quantification (I) of PDGFRβ protein levels normalized to Gapdh in whole retinal lysates of P10 wildtype and *Vtn*^−/−^ mice. n = 3 animals per genotype. Mean ± S.D.; n.s. not significant, p > 0.05; Student’s t-test.

How does vitronectin, a pericyte secreted protein regulate barrier properties in endothelial cells? One possibility is that barrier defects are due to impaired vessel development as pericytes are known to regulate endothelial sprouting and angiogenesis in the postnatal retinal vasculature (Eilken et al., 2017). However, we observed no changes in vascular patterning (Figure 5A). Specifically, we observed no changes in vessel density, capillary branching or radial outgrowth of the vasculature in *Vtn*^−/−^ mice compared to wildtype littermates (Figures 5B–D).

Another possibility is that since vitronectin is a pericyte gene and pericyte-deficient mice exhibit leaky barriers (Armulik et al., 2010; Daneman et al., 2010), barrier defects could be due to altered pericyte coverage. However, using NG2:DsRed mice that label mural cells (Figure 5E), similar pericyte coverage and pericyte density were observed in *Vtn*^−/−^ and wildtype mice (Figures 5F, 5G). PDGFRβ protein levels were also similar in *Vtn*^−/−^ and wildtype retinas (Figure 5H, 5I). Hence, vitronectin regulates barrier function without altering pericyte density or pericyte coverage.

Since vitronectin is an ECM protein, we also examined whether the barrier deficits in *Vtn*^−/−^ mice were a consequence of structural and/or functional changes in the ECM. EM analysis of retinal and cerebellum capillaries revealed no obvious structural changes in the basement membrane of endothelial cells in *Vtn*^−/−^ mice compared to wildtype mice. Moreover, immunostaining and western blotting of two highly enriched ECM proteins, collagen IV and fibronectin reveal normal localization (Figures S4A, S4B) and expression levels (Figure S4D, S4E) of these proteins in retinas of *Vtn*^−/−^ mice. Notably, we also observed normal collagen IV ensheathment of retinal blood vessels in *Vtn*^−/−^ mice (Figure S4C). Similarly, we observed normal localization and expression of two other ECM proteins, perlecan and laminin α4 in the retina vasculature of *Vtn*^−/−^ mice (Figures S4F, S4G). Finally, we also used our EM data to investigate astrocyte endfeet attachments and observed no changes in average area of cerebellum capillaries covered by astrocyte endfeet between wildtype and *Vtn*^−/−^ mice (Figure S4H, S4I). Thus, the overall structural and functional organization of the vascular basement membrane or the ECM was not compromised in mice lacking vitronectin.

Collectively, our data demonstrate that barrier defects caused in mice lacking vitronectin are not due to defects in vessel morphology and patterning, pericyte coverage, ECM organization or astrocyte endfeet. Rather, pericyte-secreted vitronectin could be acting directly on endothelial cells to regulate their barrier properties.

### Vitronectin binding to integrin receptors is essential for barrier function

**Figure 6.**
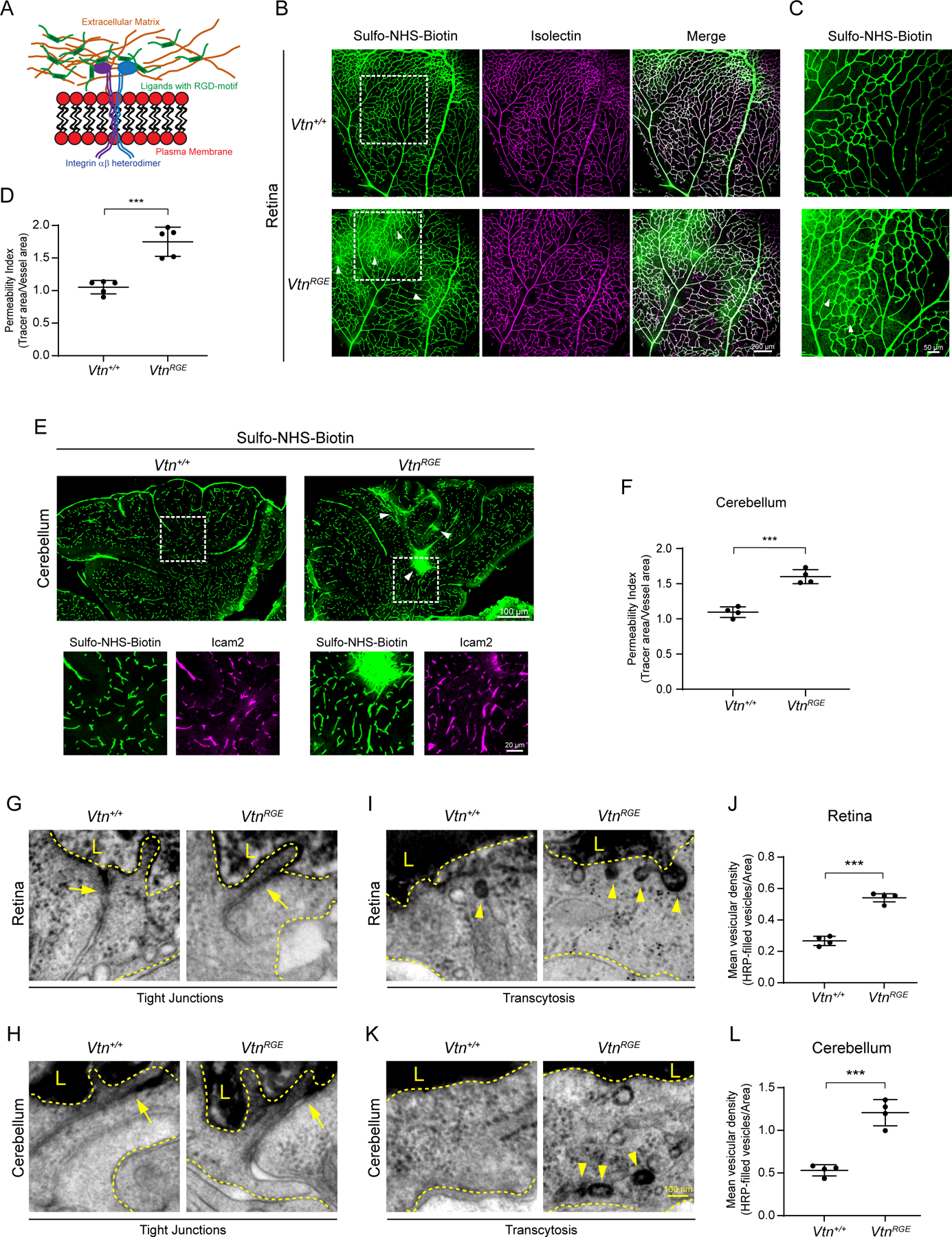
Vitronectin binding to integrin receptors is essential for barrier function (A) Schematic illustrating binding of ligands containing RGD-motif with integrin receptors (B) Leakage of Sulfo-NHS-Biotin (tracer, green) from vessels (isolectin, magenta) in P10 *Vtn*^*RGE*^ mice. White boxes correspond to higher magnification images shown in (C). (C) Tracer hotspots (arrowheads) in retinas of *Vtn*^*RGE*^ mice. (D) Quantification of vessel permeability in wildtype and *Vtn*^*RGE*^ mice. n = 5 animals per genotype. Mean ± S.D.; ***p < 0.001; Student’s t-test. (E) Leakage of Sulfo-NHS-Biotin (green) in the cerebellum of P10 *Vtn*^*RGE*^ mice. White boxes correspond to higher magnification panels showing tracer confinement to vessels (ICAM2, magenta) in wildtype mice and tracer leakage in *Vtn*^*RGE*^ mice. (F) Quantification of vessel permeability in wildtype and *Vtn*^*RGE*^ mice. n = 4 animals per genotype. Mean ± S.D.; ***p < 0.001; Student’s t-test. (G, H) EM images of HRP halting at tight junctions (arrows) in both retinas (G) and cerebellum (H) of wildtype and *Vtn*^*RGE*^ mice. Luminal (L) and abluminal sides indicated by dashed yellow lines. (I, K) EM images of HRP-filled vesicles (arrowheads) in endothelial cells of retinas (I) and cerebellum (K) of wildtype and *Vtn*^*RGE*^ mice. (J, L) Quantification of tracer-filled vesicles in endothelial cells of retinas (J) and cerebellum (L). n = 4 animals per genotype, 15–20 vessels per animal. Mean ± S.D.; ***p < 0.001, **p < 0.01; Student’s t-test.

How does pericyte secreted vitronectin signal to neighboring endothelial cells to regulate barrier permeability? Vitronectin belongs to the family of adhesion proteins that bind integrin receptors through a 3 amino acid motif, Arg – Gly – Asp (RGD) (Hynes, 2002; Preissner, 1991; Preissner and Reuning, 2011) (Figure 6A). Previous biochemical studies established that competitive binding experiments using short peptides containing the RGD domain (Orlando and Cheresh, 1991) as well as mutating vitronectin RGD to RGE (aspartic acid to glutamic acid) effectively abolished binding of vitronectin to integrins (Cherny et al., 1993). To determine if vitronectin binding to integrins is essential for barrier function, we took advantage of the knock-in mouse line harboring single point mutation of vitronectin with RGD domain mutated to RGE, *Vtn*^*RGE/RGE*^ (Wheaton et al., 2016), hereafter referred to as *Vtn*^*RGE*^.

*Vtn*^*RGE*^ mice exhibited leakage in retina and cerebellum, phenocopying *Vtn*^−/−^ mice. Similar hotspots of tracer leakage (Figure 6B) as well as neuronal cell bodies filled with tracer were apparent in the retinal parenchyma of *Vtn*^*RGE*^ mice (Figures 6C, 6D) with no obvious defects in vascular patterning. The BBB in the cerebellum of these mice was also leaky with tracer hotspots throughout the cerebellum (Figures 6E, 6F).

EM analysis of retinas and cerebellum of HRP-injected *Vtn*^*RGE*^ mice revealed similar subcellular features in endothelial cells as observed in the *Vtn*^−/−^ full knockout mice. Both the retina and cerebellum in *Vtn*^*RGE*^ mice had functional tight junctions as noted by HRP halting sharply between endothelial cells (Figures 6G, 6H). However, we observed >2-fold increase in HRP-filled vesicles in endothelial cells of *Vtn*^*RGE*^ mice compared to wildtype animals (Figures 6I, 6J, 6K, 6L), revealing upregulated transcytosis in these mice. Thus, *Vtn*^*RGE*^ mice fully recapitulate the phenotype observed in *Vtn*^−/−^ mice, indicating vitronectin binding to integrin receptors is critical for its role in regulating barrier function.

### Vitronectin-integrin α5 signaling inhibits endocytosis in CNS endothelial cells

**Figure 7.**
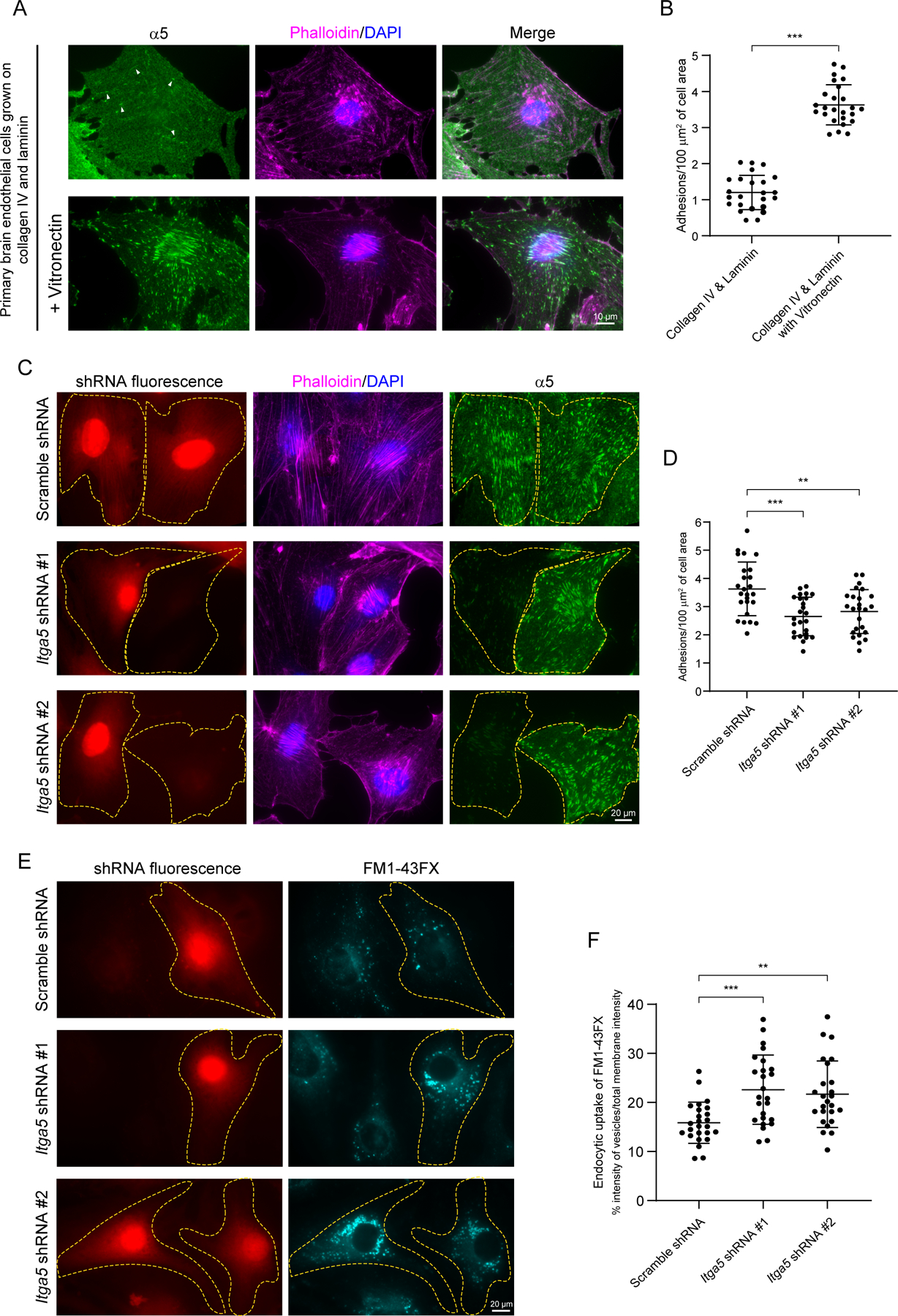
Engagement of integrin α5 with vitronectin forms adhesion structures and actively inhibits endocytosis in primary brain endothelial cells. (A, B) Representative images (A) and quantification (B) of α5 (green) containing adhesion structures in primary brain endothelial cells (phalloidin in magenta) grown on collagen IV, laminin and vitronectin-coated dishes. n = 25 cells per condition from 3 independent experiments. Mean ± S.D.; ***p < 0.001; Student’s t-test (C, D) Validation of two independent shRNAs (red) targeting endogenous α5 (green) in primary brain endothelial cells (phallodin in magenta) and quantification of adhesion structures (D) in scramble vs Itga5 shRNAs. n = 25 cells per condition from 3 independent experiments. Mean ± S.D.; ***p < 0.001, **p < 0.01; one-way ANOVA with Tukey’s post hoc test. (E) Endocytosis assay with membrane impermeable FM1-43FX (cyan) in primary brain endothelial cells transfected with shRNAs (red) targeting endogenous α5. (F) Quantitation of endocytic uptake of FM1-43FX. n = 25 cells per condition from 3 independent experiments. Mean ± S.D.; ***p < 0.001, **p < 0.01; one-way ANOVA with Tukey’s post hoc test.

What are the specific integrin receptors on endothelial cells required for vitronectin-mediated regulation of barrier function? Of the several α and β integrin subunits that heterodimerize to form integrin receptors (Hynes, 2002) we focused on the two α receptors, α5 and αv that have been shown to interact with RGD-ligands. While αv is considered the predominant receptor for vitronectin, cell motility studies have also demonstrated interaction of vitronectin with α5 (Bauer et al., 1992; Zhang et al., 1993). Indeed, when primary mouse brain endothelial cells were grown on vitronectin-coated dishes, we readily observed adhesive structures containing endogenous integrin α5 (Figures S5A). In contrast, no adhesion structures were observed in cells grown on collagen IV coated or laminin-511 (α5β1γ1, hereafter referred to as laminin) coated dishes, demonstrating specific binding and activation of integrin α5 by vitronectin (Figures S5A, S5B). To mimic and recapitulate the *in vivo* ECM as much as possible we also grew these cells on a combination of ECM ligands. Similar to our findings with individual ligands, we observed integrin α5 containing adhesion structures only in the presence of vitronectin (Figures 7A, 7B). Furthermore, >95% of α5 adhesions were positive for and co-localized with classic focal adhesion proteins such as phosphorylated FAK (Y397), paxillin, and vinculin (Figures S5C, S5D), indicating that α5 adhesions are indeed bonafide focal adhesion structures. Thus, vitronectin actively engages integrin α5 receptors in CNS endothelial cells and drives the formation of α5 containing focal adhesions.

Since deletion of vitronectin results in barrier leakage due to increased transcytosis, we next directly investigated the role of vitronectin-integrin α5 interactions in vesicular trafficking in primary brain endothelial cells. Knockdown of integrin α5 in these cells with two independent shRNAs (Figure 7C, 7D), resulted in a significant increase in endocytosis (Figures 7E, 7F). In cells depleted of integrin α5 by either shRNA, FM1-43FX dye was readily incorporated in the newly formed vesicles that were endocytosed from plasma membrane, whereas very few FM1-43FX dye-positive vesicles were observed within the cells transfected with scrambled shRNA. These results demonstrate that the engagement of abluminal vitronectin-integrin α5 actively inhibits endocytosis in CNS endothelial cells.

### Integrin receptor, α5, is required in endothelial cells for blood-CNS barrier integrity

**Figure 8.**
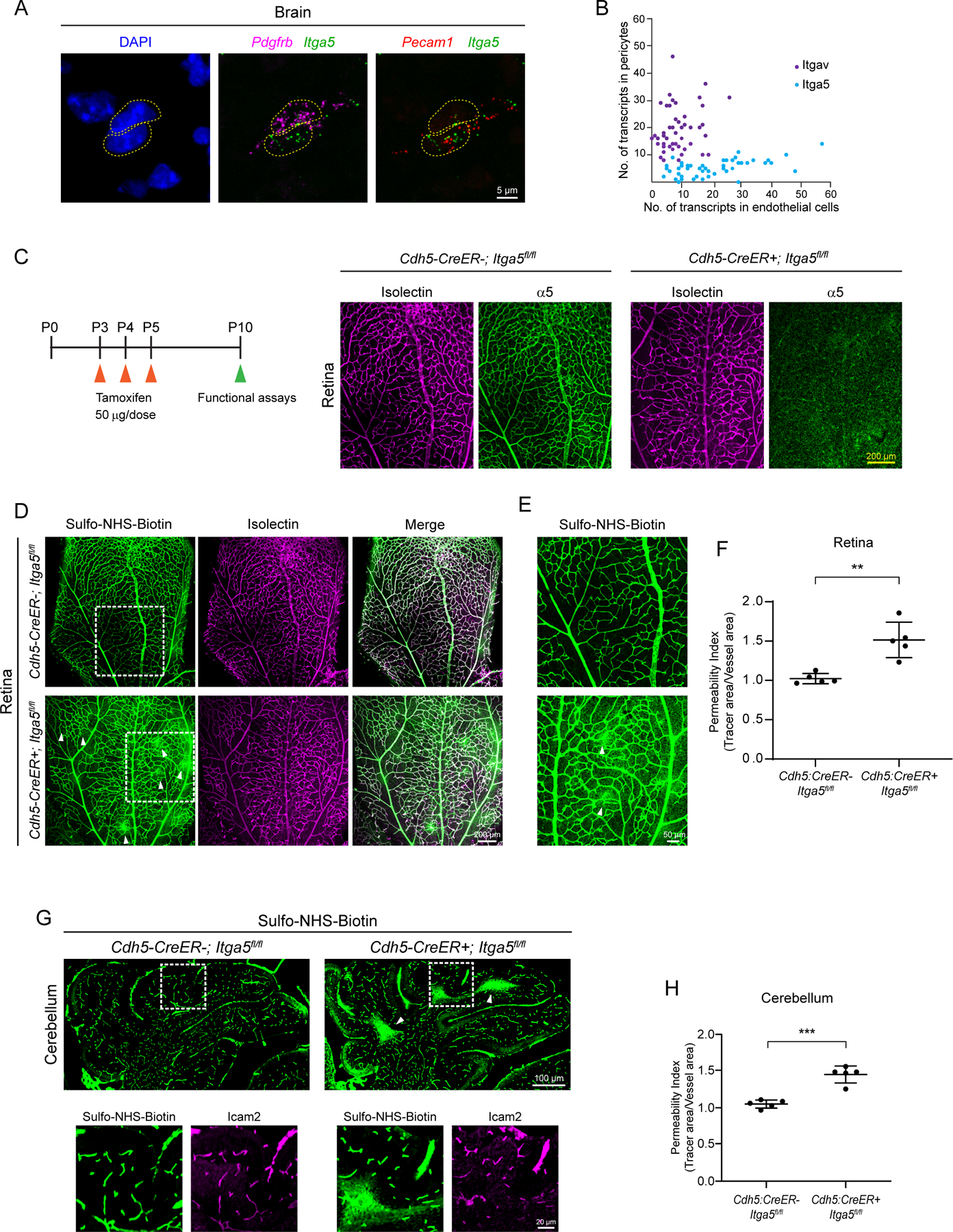
Integrin α5 in endothelial cells is specifically required for blood-CNS barrier function (A) In situ hybridization for *Itga5* (green), *Pecam1* (endothelial gene, red) and *Pdgfrb* (pericyte gene, magenta) in P7 brain tissue. (B) Scatter plot of *Itga5* (blue) and *Itgav* (purple) transcript numbers in pericytes versus endothelial cells from RNAscope in situ hybridization as shown in (A). Each dot represents an individual pericyte-endothelial cell pair, n = 49 and 47 cell pairs respectively from 3 animals. (C) Depiction of tamoxifen injections in postnatal pups from P3-P5 and validation of tamoxifen-induced deletion of α5 (green) from vessels (isolectin, magenta) in P10 mice. (D) Sulfo-NHS-Biotin (tracer, green) leaks out of vessels (isolectin, magenta) in P10 *Cdh5:CreER+; Itga5*^*fl/fl*^ mice. Tamoxifen administered P3-P5, see Figure S6. White boxes correspond to higher magnification images in (E). (E) Tracer hotspots (arrowheads) in mice lacking endothelial Itga5. (F) Quantification of vessel permeability in wildtype and *Cdh5:CreER+; Itga5*^*fl/fl*^ mice. n = 5 animals per genotype. Mean ± S.D.; **p < 0.01; Student’s t-test. (G) Sulfo-NHS-Biotin (green) leakage in cerebellum of P10 *Cdh5:CreER+; Itga5*^*fl/fl*^ mice. White boxes correspond to higher magnification images with tracer and vessels (ICAM2, magenta). (H) Quantification of vessel permeability in wildtype and *Cdh5:CreER+; Itga5*^*fl/fl*^ mice. n = 5 animals per genotype. Mean ± S.D.; ***p < 0.001; Student’s t-test.

To identify the relevant endothelial integrin receptors *in vivo*, we first performed *in situ* hybridization to examine the localization of *Itga5* and *Itgav* transcripts in the brain. Single cell RNA-seq data reveals transcripts for both genes in CNS endothelial cells (Vanlandewijck et al., 2018); α5 and αv are also known to co-operate during vascular development (Van Der Flier et al., 2010). RNAscope reveals that although both *Itga5* and *Itgav* mRNA are present in endothelial cells as well as pericytes in the brain (Figure 8A, S7A), *Itga5* mRNA is predominantly expressed in endothelial cells whereas *Itgav* mRNA is predominately expressed in pericytes (Figure 8B). On average, *Itga5* transcripts in endothelial cells was 20.3 ± 1.5 and in pericytes it was 5.3 ± 0.43. In contrast, the average *Itgav* transcripts in endothelial cells was 8.3 ± 0.7 and in pericytes it was 19.5 ± 1.2 (Figure 8B, Mean ± SEM). Thus, *Itga5* is predominantly expressed in endothelial cells while *Itgav* is predominantly in perciytes.

We next determined the role of endothelial *Itga5* and *Itgav* in barrier function by ablating these genes acutely and specifically in endothelial cells by crossing *Itga5*^*fl/fl*^ or *Itgav*^*fl/fl*^ (Van Der Flier et al., 2010) mice with endothelial cell-specific *Cdh5-CreER* mouse line (Wang et al., 2010). Acute deletion of endothelial *Itga5* (Figure 8C) resulted in a leaky blood-retinal barrier (Figure 8D) with numerous tracer hotspots spread out in the retina tissue (Figures 8E, 8F). Importantly, *Itga5*^*fl/fl*^*;Cdh5-CreER* mutant mice exhibited normal vascular density and patterning (Figure S6A–S6D). Similar to *Vtn*^−/−^ and *Vtn*^*RGE*^ *mice*, *Itga5*^*fl/fl*^*;Cdh5-CreER* mutants also had a leaky BBB in the cerebellum (Figures 8G, 8H). In contrast to *Itga5*, mice lacking endothelial *Itgav* exhibited normal barrier function. The injected tracer was completely confined to the vasculature in both the retina (Figures S7B–S7D) and the brain tissue (Figures S7E, S7F) indicating intact blood-CNS barriers in *Itgav*^*fl/fl*^*;Cdh5-CreER* mice. These experiments establish the RGD-specific integrin receptor, α5 as an essential integrin for barrier function, particularly in the retina and cerebellum. Importantly, mice lacking endothelial integrin α5 receptor fully phenocopy *Vtn*^−/−^ and *Vtn*^*RGE*^ mice. Together with our *in vitro* results, these data indicate that the engagement of vitronectin-integrin α5 actively inhibits endocytosis in CNS endothelial cells to ensure barrier integrity. These results demonstrate that ligand-receptor interactions between pericyte-derived vitronectin and endothelial integrin, α5, are critical for barrier integrity by actively inhibiting transcytosis in CNS endothelial cells *in vivo*.

## Discussion

In this study, we establish how pericytes signal to endothelial cells via vitronectin-integrin interactions to maintain low rates of transcytosis, thus, ensuring barrier integrity. We identify and demonstrate an essential role for pericyte-secreted vitronectin in blood-CNS barrier function. Vitronectin deposited in the extracellular matrix, binds to integrin receptors on CNS endothelial cells and this interaction suppresses transcytosis, thus, regulating barrier integrity. Lack of vitronectin or mutating vitronectin to prevent integrin binding as well as endothelial-specific integrin deletion – all of these genetic manipulations result in dysfunctional blood-CNS barriers, highlighting the role of ligand-receptor interactions of pericyte-to-endothelial signaling in barrier function.

Although liver-derived vitronectin is found in the plasma at high levels, our *in vivo* siRNA experiments demonstrate that plasma vitronectin is likely dispensable for barrier function. Moreover, integrin receptors are found to be enriched on the abluminal endothelial membranes compared to luminal membranes (Izawa et al., 2018; Del Zoppo and Milner, 2006) and our experiments in primary brain endothelial cells where vitronectin is only present on the abluminal side shows that abluminal vitronectin is sufficient to form adhesion structures and is necessary to inhibit endocytosis via interactions with integrin α5 receptor. Thus, our data establish that it is the pericyte-derived vitronectin in CNS that is required for barrier function.

How does pericyte-derived vitronectin interacting with integrin receptors actively inhibit endocytosis in CNS endothelial cells? The adhesive forces generated by the engagement of integrins have been shown in various cell types to maintain plasma membrane tension (Katsumi et al., 2004; Ross et al., 2013). Disengaging integrin receptors from vitronectin likely causes a reduction in membrane tension and it is well known that decreased membrane tension promotes increased endocytosis (Diz-Muñoz et al., 2013; Sheetz and Dai, 1996; Thottacherry et al., 2018). Thus, it is plausible that vitronectin binding to integrin α5 exerts adhesive forces to maintain the plasma membrane tension to ensure low rates of transcytosis in CNS endothelial cells. Our results indicate that signals from perivascular cells to endothelial cells via designated ligand-receptor pairs contribute to the unique biophysical properties of CNS endothelial membrane for barrier integrity.

Our work demonstrates the importance of signaling between perivascular cells and CNS endothelial cells in modulating blood-CNS barriers. It is likely that in addition to vitronectin, other ECM molecules also play key roles in barrier function given that ECM is a hub for ligand-receptor interactions across multiple cell types. Indeed, there is evidence for fibronectin interacting with α5 receptor impacting brain endothelial cell survival (Wang and Milner, 2006) and promoting blood-brain barrier integrity following stroke (Wang et al., 2019a). Absence of integrin receptors also triggers onset of experimental autoimmune encephalomyelitis (Kant et al., 2019). Not surprisingly, ECM breakdown is a hallmark feature associated with many diseases and disorders of the CNS (Thomsen et al., 2017). Thus, our findings reveal new molecular targets and pathways within the CNS for development of novel therapeutics that could aid CNS drug delivery.

## RESOURCE AVAILABILITY

### Lead Contact

Further information and requests for resources and reagents should be directed to and will be fulfilled by the Lead Contact, Chenghua Gu (chenghua_gu@hms.harvard.edu)

### Materials Availability

This study did not generate new unique reagents

### Data and Code Availability

- All data are available in the manuscript or supplemental information.
- The codes used have been previously published and are freely available (see Key Resources table).
- Any additional information required to reanalyze the data reported in this work paper is available from the Lead Contact upon request.

## STAR METHODS

### EXPERIMENTAL MODEL AND SUBJECT DETAILS

#### Mice

All mouse experiments were performed according to institutional and US National Institutes of Health (NIH) guidelines approved by the International Animal Care and Use Committee (IACUC) at Harvard Medical School. Mice were maintained on 12 light/12 dark cycle.

The following mice strains were used in this study: Both *Vtn* null mice (JAX: 004371) and *Vtn*^*RGE/RGE*^ mice (Wheaton et al., 2016) were generous gifts from Dr. Thomas Sisson, University of Michigan; *Cdh5-CreER* was kindly provided by Dr. Ralf Adams, Max-Planck Institute of Molecular Biomedicine; *NG2:DsRed* (JAX: 008241), *Itga5*^*flox*^ (JAX: 032299), *Itgav*^*flox*^ (JAX: 032297); were obtained from Jackson Laboratories. All mice were maintained on C57/BL6J background. All leakage assays were performed at P10, RNAscope was performed at P7. *In vivo* siRNA experiments were done in 6-week old mice.

Cre-mediated recombination was induced by intraperitoneal injection of 50 ug of 1 mg/ml Tamoxifen (T5468, Sigma dissolved in peanut oil containing ethanol 1:40 by volume) for 3 consecutive days, P3-P5. All animals were genotyped with allele-specific PCR reactions prior to experiments. Both males and females were used across all experiments and no sex-dependent phenotype was observed.

### METHOD DETAILS

Across all mouse experiments, mice were genotyped the day of experiments and experiments were not done genotype-blinded. However, all analyses were done blinded. No statistical methods were used to predetermine samples sizes but our sample sizes are consistent with previous publications and standards in the field.

#### Immunohistochemistry

Enucleated eyes of P10 pups were fixed in 4% ice-cold PFA (EMS, 15713) for 5 minutes at room temperature (RT). Retinas were then dissected in 4% PFA and allowed to fix for 30 minutes at RT (adult retinas were fixed for 1 hour at RT). Retinas were washed in PBS three times for 5 minutes each and incubated in blocking buffer (10% normal donkey serum with 5% bovine serum albumin and 0.5% triton in PBS) for 1 hour at RT. Retinas were then incubated with primary antibodies in blocking buffer overnight at 4°C. The next day retinas were washed again with PBS three times for 5 minutes each and incubated with secondary antibodies in blocking buffer for 1 hour at RT and washed again with PBS. Retinas were then dissected into 4 leaflets and flat mounted on glass slides with vitreal surface in contact with coverslips using Prolong gold Antifade mountant (Thermo Fisher Scientific P36934)

Dissected brains were drop-fixed in 4% PFA overnight at 4°C. The brains were washed with PBS three times 10 minutes each and cryopreserved in 30% sucrose. The brains were bisected along the midline to obtain sagittal sections, before freezing in TissueTek OCT (Sakura). 20 um cryosections obtained on the cryostat were then processed for immunostaining. Brain sections were first permeabilized with 0.2% triton in blocking buffer (10% normal donkey serum with 5% bovine serum albumin in PBS) for 10 minutes at RT. The rest of the staining procedure was similar to the stainings in retina as above, except secondary antibodies were incubated for 45 minutes at RT.

Isolectin GS-IB_4_ conjugated to Alexa Fluor 568 (Thermo Fisher Scientific I21412) or Isolectin GS-IB_4_ conjugated to Alexa Fluor 647 (Thermo Fisher Scientific I32450) was incubated (1:300) along with primary antibodies to stain vasculature in retinas. Streptavidin conjugated to Alexa Fluor 647 (Thermo Fisher Scientific S32357) was incubated (1:300) along with secondary antibodies to detect the injected Sulfo-NHS-Biotin. The following primary antibodies were used: rabbit α-Vitronectin (Genway Biotech GWB-794F8F, 1:100), goat α-CD31 (PECAM1, R&D Systems AF3628, 1:100), mouse α-Claudin-5 (Thermo Fisher Scientific 352588, 1:200), rabbit α-ZO-1 (Invitrogen 40–2200, 1:200), rat α-CD102 (ICAM2, BD Biosciences 553326, 1:100), rabbit α-ERG1/2/3 (Abcam ab92513, 1:200), rabbit α-Collagen IV (Bio-rad 2150–1470, 1:200), rat α-Perlecan (EMD Millipore, MAB1948P, 1:200), goat α-laminin α4 (R&D systems, AF3837, 1:100) rat α-CD49e (α5, BD Biosciences 553319, 1:100). All corresponding secondary antibodies were used at 1:300 obtained from Jackson Immunoresearch Laboratories.

#### Fluorescent in situ hybridization

Brains and lungs were dissected from 1-week old wildtype animals, flash frozen in liquid nitrogen and cryosectioned to obtain 20 um sections. RNAscope was performed according to manufacturer’s instructions (ACD Bio) with the following probes: *Vtn* (443601), *Pdgfrb* (411381-C3), *PECAM1* (316721-C2), *Itga5* (575741) *Itgav* (513901). To perform immunostaining post RNAscope, slides were briefly rinsed in PBS and incubated with blocking buffer (3% normal donkey serum with 3% bovine serum albumin in PBS) for 1 hour at RT. The rest of the steps for staining are similar to the staining protocol mentioned above under immunohistochemistry. DAPI (Thermo Fisher Scientific 62247) was added at 1:5000 dilution in the last PBS wash before mounting slides.

#### Barrier permeability assays

P10 pups were briefly anaesthetized with 3% isoflurane. Eyelid of one eye was cut off and tracer was injected retro-orbitally (Yardeni et al., 2011) with a 30-gauge needle. Tracer was allowed to circulate for 5 minutes followed by dissection of contralateral retina and brain, and processed for immunohistochemistry.

Tracers include EZ-Link Sulfo-NHS-LC-Biotin (Thermo Fisher Scientific, 21335, 0.44 kDa) injected at 0.5 mg/gm body weight and 10 kDa Dextran conjugated Alexa 488 (Thermo Fisher Scientific, D22910) injected at 0.2 mg/gm body weight. Tracers were made fresh in PBS, dissolved in 5 ul of PBS/gm body weight.

#### *In vivo* siRNA experiments

250 nmol of Ambion’s HPCL-IVR (HPLC grade, *in vivo* ready) pre-designed siRNAs targeting mouse vitronectin (Catalog No. 4457308, siRNA IDs s76001, s76002) and a control siRNA (Catalog No. 4457289) were ordered from ThermoFisher. Invivofectamine 3.0 (Thermo Fisher, IVF3001) was used to form siRNA complexes, as per manufacturer’s instructions for systemic delivery into mice. siRNA duplexes were resuspended in DNase/RNase free water (Thermo Fisher, 10977) at 4.8 mg/ml, the complexation in Invivofectamine 3.0 was followed as per protocol. 100 ul of siRNA complex was injected into 6-week old mice (~20 gm) to result in 1 mg/kg dosing. siRNA complexes were injected into circulation through tail vein on 2 consecutive days. Subsequent experiments included confirmation of knock-down and leakage assays which were performed either 24 hours (day 3) or 72 hours (day 5) post last siRNA injection. Confirmation of vitronectin knock-down was determined by ELISA on plasma isolated (see below) from mice. For leakage assays, Sulfo-NHS-biotin was injected (0.5 mg/gm body weight) into circulation via the tail-vein. Across all mice, retinas from left eyes were used for leakage analyses.

#### Plasma isolation

On the day of experiments, mice were briefly anaesthetized with 3% isoflurane and heparinized capillary tubes 1.1 mm × 7.5 mm (Thomas Scientific, 44B508) were used to collect blood from the retro-orbital sinus of the right eye. Collected blood was transferred to Eppendorf tubes containing 10 ul 0.5 M EDTA, pH 8.0 (Thermo Fisher, 15575020) and vortexed to prevent blood from coagulating. Isolated blood samples were centrifuged for 15 minutes at 2000xg at 4°C and the supernatant was collected. Knockdown of vitronectin in plasma samples was confirmed by an ELISA kit for vitronectin (Molecular Innovations, MVNKT-TOT).

#### Transmission electron microscopy

HRP (Thermo Fisher Scientific, 31491), prepared fresh before each experiment was injected into the retro-orbital sinus of P10 pups briefly anaesthetized with isoflurane. HRP was injected at 0.5 mg/gm body weight, dissolved in 5 ul of PBS/body weight. Tracer was circulated for 10 minutes followed by enucleation of the contralateral eye and retina dissection in 4% PFA made in 0.1 M sodium cacodylate buffer (EMS, 11653). The brain was also dissected and both the tissues were first fixed in 5% glutaraldehyde (EMS, 16200) and 4% PFA mixture made in 0.1 M sodium cacodylate for 1 hour at RT. The brains and retinas were then fixed overnight at 4 °C in 4% PFA in 0.1 M sodium cacodylate.

Following fixation both tissues were washed with 0.1 M sodium cacodylate buffer three times, 5 minutes each. 100 um sagittal sections of the brain and the whole retina were then incubated with fresh 0.5 mg/ml DAB (Sigma, D5905) for 25 min at RT. DAB was prepared in 0.1 M sodium cacodylate containing 0.05 M Tris-HCl and 0.01% hydrogen peroxide. Leaky areas within the retina and cerebellum were further microdissected, cut into 80 nm ultrathin sections and further processed for EM analysis as described previously (Chow and Gu, 2017).

#### Western Blotting

Retinas were dissected in PBS containing protease inhibitors and phosphatase inhibitor (Thermo Fisher Scientific, 87786 and 78420 respectively). Dissected retinas were further minced with a razor blade and allowed to lyse on ice for 30 minutes in lysis buffer (50 mM Tris-HCl, pH 7.4, 150 mM NaCl, 1% triton-X 100, 0.1% SDS supplemented with protease and phosphatase inhibitors). Lysed retinas were centrifuged for 15 minutes at 4°C, the supernatant was collected and BCA assay (Thermo Fisher Scientific, 23225) was performed according to manufacturer’s instructions to determine total protein concentrations. Samples were then analyzed for various proteins using SDS-PAGE and Western Blotting. The following antibodies were used: Pdgfrβ (Cell Signaling Technologies 3169, 1:500), Fibronectin (Abcam ab2413, 1:200)

#### Cell culture media, growing conditions and coverslip coating

Mouse primary brain microvascular endothelial cells (Cell Biologics, C57-6023) were maintained in a complete mouse endothelial cell medium (Cell Biologics, M1168) supplied with VEGF, ECGS, Heparin, EGF, and FBS, according to the manufacture’s instruction. To functionalize the surface of cover glasses, we first cleaned the cover glasses (Thomas Scientific, 1217N79) with air plasma (Harrick Plasma) for 15 min. For surface functionalization with single matrix proteins, the cleaned cover glasses were incubated with 50 μg/mL mouse collagen IV (Corning, 354233) in water, 50 μg/mL laminin 511 (Sigma-Aldrich, CC160) in PBS, or 50 μg/mL multimeric vitronectin (Molecular Innovations, HVN-U) in PBS, for 1 h at 37°C. For surface functionalization with collagen IV plus laminin, the collagen IV-functionalized cover glasses were air dried and then incubated with 50 μg/mL laminin 511 in PBS for 1 h at 37°C. For surface functionalization with Collagen IV plus laminin and vitronectin, the collagen IV-functionalized cover glasses were air dried and then incubated with 50 μg/mL laminin 511 and vitronectin in PBS for 1 h at 37°C. The functionalized cover glasses were washed with cell culture media 3 times before cell plating. Cells were gently detached from tissue culture dishes using enzyme-free cell dissociation buffer (Gibco, 13151014) and then seeded on the functionalized cover glasses for at least 4 h before next treatment.

#### Imaging focal adhesions in cell culture

To image integrin α5-mediated adhesion, the cells were snap chilled in ice-cold HHBSS, which is 1X HBSS (Gibco, 24020117) buffered with 10 mM HEPES (Gibco, 15630106). The cells were then incubated with primary antibody rat α-CD49e (α5, BD Biosciences 553319, 1:100) in HHBSS for 30 min at 4°C and washed with ice-cold HHBSS 3 times for 5 min each. The cells were fixed in 4% ice-cold PFA in BPS for 15 min at RT and permeabilized with 0.1% triton-X in PBS for 15 min at RT. The cells were then washed with PBS 3 times for 5 min each and incubated in blocking buffer (5% bovine serum albumin in PBS) for 1 hour at RT. To simultaneously image paxillin, vinculin or pFAK with integrin α5, the blocked samples were incubated with corresponding primary antibody in blocking buffer for 2 h at RT and washed with PBS 3 times for 5 min each. Primary antibodies include mouse α-paxillin (BD Biosciences 610051, 1:100), mouse α-vinculin (Sigma-Aldrich V9131, 1:100) and rabbit α-FAK (phosphor Y397 from Abcam ab81298, 1:100). The samples were finally incubated with corresponding secondary antibodies at 1:500, phalloidin-iFluor 488 (Abcam, ab176753), and nuclear stain which is 1 μg/mL of either Hoechst 33342 (Thermo Scientific, 62249) or Ethidium Homodimer-1 (Thermo Scientific, E1169) in blocking buffer for 1 h at RT and washed with PBS 5 times for 5 min each before imaging.

#### *In vitro* shRNA and endocytosis experiments

The DNA sequences encoding shRNA1 and 2 for integrin α5 knockdown are “CCCAGCAGGGAGTCGTATTTACTCGAGTAAATACGACTCCCTGCTGGG” and “ATCAACTTGGAACCATAATTACTCGAGTAATTATGGTTCCAAGTTGAT”, respectively. The DNA sequence encoding negative control or scramble shRNA is “CCTAAGGTTAAGTCGCCCTCGCTCGAGCGAGGGCGACTTAACCTTAGG”, which is designed based on previously published scramble shRNA plasmid (from David Sabatini, addgene plasmid # 1864). These DNA fragments were cloned into pLKO.1 (a gift from David Root, Addgene plasmid # 10878) for RNA transcription in cells. The DNA encoding EBFP2 was cloned into the pLKO.1 vector in substitution of the sequence encoding neomycin resistant protein to report the transcription of shRNA. To package lentivirus for RNAi, HEK293FT cells (Invitrogen, R70007) in 35-mm tissue culture dishes were transfected with 1.5 μg pLKO.1 shRNA plasmid, 0.8 μg psPAX2 (a gift from D. Trono, Addgene plasmid # 12260), 0.7 μg pMD2.G (a gift from D. Trono, Addgene plasmid # 12259), and 9 μL Turbofect transfection reagent (Thermo Scientific, R0533) in 2 mL of Opti-MEM (Gibco, 31985062) for 12 h. The viruses were produced in DMEM supplied with 10 % FBS and 110 mg/mL sodium pyruvate (Gibco) and harvested 24 h after transfection. The mouse primary brain endothelial cells were infected with lentivirus and used for experiments 3 days after infection.

To test endocytosis, cells were washed with HHBSS 3 times and starved in HHBSS for 1 h at 37°C. The cells were then incubated with 5 μg/mL of FM™ 1-43FX (Thermo Fisher Scientific, F35355) in HHBSS for 15 min at 37°C and then quickly washed with HHBSS 5 times. The cells were immediately fixed in 4% PFA in PBS for 15 min at RT and washed with PBS 3 times before imaging.

#### Imaging

Leakage assays in brain tissue were imaged on Olympus VS120 slide scanner. Rest of the fluorescent imaging was acquired on Leica TCS SP8 confocal. Z-stacks were obtained and all maximum-intensity projections are shown in all figures. TEM images were acquired on a 1200EX electron microscope (JOEL) equipped with a 2k CCD digital camera (AMT). All images were processed using FIJI. Cell culture imaging was acquired on an epi-fluorescence Leica DMI 6000B microscope.

### QUANTIFICATION AND STATISTICAL ANALYSIS

Except for scatter plots, all graphs represent mean ± S.D. All graphs show individual data points throughout the paper. For each graph, the sample size and statistical test used are described in the corresponding figure legend. No method was used to predetermine whether the data met assumptions of the statistical approach. For mouse experiments n is the number of mice whereas for *in vitro* experiments n is the number of cells.

#### Fluorescent In situ hybridization

To determine percentage of pericytes positive for *Vtn* mRNA transcripts, PECAM1 immunostaining was first used to determine if cells positive for *Pdgfrb* transcripts were pericytes. Vessels containing only a single nucleus and ≤ 5 μm in width as shown in Figure 1C were identified as capillaries and *Pdgfrb*+ cells in close-contact or abutting the vessel were identified as pericytes (Figure 1C).

Scatter plots for *Pdgfrb* vs *Vtn* mRNA puncta was obtained by first identifying nuclei using the DAPI channel. DAPI containing nuclei were first segmented using FIJI and segmented regions were recorded in ROI manager. Fluorescent images of *Pdgfrb* and *Vtn* were thresholded using ‘Otsu’ method. *Pdgfrb* and *Vtn* puncta numbers in each of the segmented regions were obtained from the thresholded images using ‘Find Maxima’ with tolerance ≥ 30. Puncta numbers obtained for both transcripts in a given cell were plotted as X-Y scatter.

Scatter plot for *Itga5* or *Itgav* mRNA transcripts in endothelial cells vs pericytes was obtained in a similar manner. However, the DAPI nuclei segmented were first assigned as endothelial cells or pericytes using *Pecam1* and *Pdgfrb* mRNA respectively.

#### Leakage assays

Leakage was quantified as previously described (Andreone et al., 2017; Chow and Gu, 2017). For retinas, 770 μm × 770 μm areas across all 4 leaflets were first maximum intensity Z-projected, background subtracted, thresholded to obtain area of vessel (isolectin for retinas and ICAM2 for brain sections) and tracer (Sulfo-NHS-Biotin). ‘Default’ threshold method was used for thresholding isolectin signal while ‘Li’ thresholding was used for streptavidin (tracer) signal.

Permeability index was defined as the ratio of area of tracer to area of vessel. Permeability index value of 1 implies tracer confined within vessels while values >1 indicate tracer extravasation from vessels. For leakage analysis of brain tissues, 6–8 sections were quantified per animal. Average of values across these regions/sections for a given animal was treated as one biological sample.

#### ELISA for vitronectin protein expression levels

ELISA analyses were performed using Arigo Biolaboratories free ELISA calculator at https://www.arigobio.com/elisa-analysis. Standard curve was generated by fitting the data to a 4-parameter logistic (4PL) curve. Our preliminary ELISA runs determined that plasma samples diluted 1:500 were usually in the range of the standard curve.

#### Vesicular density from EM data

For both retina and cerebellum, 15–20 vessels per animal were imaged under an electron microscope. Low magnification images encompassing complete blood vessel was first acquired using which the entire cytoplasmic area of each vessel was determined. For every blood vessel, tracer-filled vesicles were manually counted and then normalized to cytoplasmic area of the vessel (excluding area of nucleus) to obtain vesicular density. Average of vesicular density across all vessels of a given animal was considered one biological sample.

#### Vessel density, branching, radial outgrowth

Tilescan images to capture entire leaflets of retinas were acquired and these parameters were obtained for each leaflet. The average of all 4 leaflets was taken to be the measurement for a given animal. For vessel density measurements, isolectin stained vessel images were auto-thresholded to determine vessel area. Ratio of vessel area was normalized to total leaflet area to obtain vessel density measurements. For capillary branches, capillary branch points were counted manually in multiple 300 μm × 300 μm regions per leaflet, and the average across these regions was obtained for each animal. For P10 retinas, 300 μm × 300 μm regions were ideal as it allowed for the exclusion of arteries and veins. For radial outgrowth, vessel images were first auto-thresholded, distance from the optic nerve head to the tip of the retina was measured and this was normalized to distance from optic nerve head to edge of retinal tissue.

#### Pericyte density and coverage

Pericyte density and coverage analyses were performed as described previously (Chow and Gu, 2017). 770 μm × 770 μm areas across all 4 leaflets were first maximum intensity Z-projected and background subtracted. Erg and isolectin were auto-thresholded across all images and genotypes. For pericyte density measurements, DsRed signal was thresholded using ‘Otsu’ method to identify only pericyte cell bodies. The number of DsRed+ cell bodies was normalized to endothelial cell nuclei count to determine pericyte density. For pericyte coverage measurements, DsRed signal was thresholded using ‘Li’ method to allow detection of pericyte cell bodies as well as processes. Area of DsRed obtained was then normalized to area of thresholded isolectin to determine pericyte coverage.

#### Astrocyte endfeet coverage from EM data

Low magnification EM images were acquired to capture entire cross-section of vessels. The vascular basement membrane was first identified in each vessel (encompassing endothelial cells and pericytes) and perimeter of the cross-section was first obtained. Astrocyte endfeet coverage was defined as vessel perimeter in contact with astrocyte endfeet normalized to total perimeter of the vessel cross-section. Average of percent coverage of all vessels from one animal was reported as the percent coverage for that mouse.

#### Western Blots

All western blots were quantified using the gel analyzer tool in Fiji. Sample values were normalized to GAPDH values from corresponding lanes to account for differences in protein amounts across wells. The ratio of band intensity values of lysates from *Vtn*^−/−^ mice to wildtype mice was then obtained to plot fold-change for mutant mice.

#### Focal adhesions and dye uptake assays

All images were processed using ImageJ Fiji. To identify integrin α5-mediated adhesions, the images were first background subtracted and thresholded. The particle analysis tool in Fiji was then used to quantify the number of adhesions in all imaging channels. The bright endocytic vesicles were identified by threshold tool and the ratio (%) of total fluorescence intensity in endocytic vesicles to the whole cell fluorescence intensity was measured to quantify endocytosis.

#### Statistical Analysis

All statistical analyses were performed using Prism 9 GraphPad software. For comparison between two groups, unpaired two-tailed Student’s t-test was used. For comparison across multiple groups, one-way ANOVA with Tukey’s post-hoc test was used.

## Supplementary Material

2

## Figures and Tables

**KEY RESOURCES TABLE:** 

REAGENT or RESOURCE	SOURCE	IDENTIFIER
**Antibodies**		
Rabbit polyclonal α-Vitronectin	Genway Biotech	Cat# GWB-794F8F; RRID:AB_10287185
Goat polyclonal α-PECAM1	R&D Systems	Cat# AF3628; RRID:AB_2161028
Mouse monclonal α-Claudin-5 488 conjugate	Thermo Fisher Scientific	Cat# 352588, RRID:AB_2532189
Rabbit polyclonal α-ZO-1	Thermo Fisher Scientific	Cat# 40–2200, RRID:AB_2533456
Rat monoclonal α-CD102	BD Biosciences	Cat# 553326, RRID:AB_394784
Rabbit monoclonal α-ERG½/3	Abcam	Cat# ab92513, RRID:AB_2630401
Rabbit polyclonal α-Collagen IV	Bio-Rad	Cat# 2150–1470, RRID:AB_2082660
Rat monoclonal α-Perlecan	Millipore	Cat# MAB1948P, RRID:AB_10615958
Goat polyclonal α-laminin α4	R&D Systems	Cat# AF3837, RRID:AB_2249744
Rat monoclonal α-CD49e	BD Biosciences	Cat# 553319, RRID:AB_394779
Rabbit monoclonal α-PDGFRβ	Cell Signaling Technology	Cat# 3169
Rabbit polyclonal α-Fibronectin	Abcam	Cat# ab2413, RRID:AB_2262874
Mouse monoclonal α-Paxillin	BD Biosciences	Cat# 610051, RRID:AB_397463
Mouse monoclonal α-Vinculin	Sigma-Aldrich	Cat# V9131, RRID:AB_477629
Rabbit monoclonal α-FAK (phospho Y397)	Abcam	Cat# ab81298, RRID:AB_1640500
**Chemicals, Peptides, and Recombinant Proteins**		
Isolectin GS-IB4	Thermo Fisher Scientific	Cat# I21412; Cat# I32450
Streptavidin conjugated to Alexa Fluor 647	Thermo Fisher Scientific	Cat# S32357
EZ-link Sulfo-NHS-C-Biotin	Thermo Fisher Scientific	Cat# 21335
10 kDa Dextran conjugated to Alexa Fluor 488	Thermo Fisher Scientific	Cat# D22910
Horseradish Peroxidase	Thermo Fisher Scientific	Cat# 31491
Invivofectamine 3.0	Thermo Fisher Scientific	Cat# IVF3001
3, 3 - Diaminobenzidine tetrahydrochloride	Sigma-Aldrich	Cat# D5905
Collagen IV	Corning	Cat# 354233
Laminin-511	Sigma-Aldrich	Cat# CC160
Vitronectin	Molecular Innovations	Cat# HVN-U
Phalloidin-iFLuor 488	Abcam	Cat# ab176753
FM 1–43FX	Thermo Fisher Scientific	Cat# F35355
**Critical Commercial Assays**		
ELISA kit for Vitronectin	Molecular Innovations	Cat# MVNKT-TOT
**Experimental Models: Cell Lines**		
Mouse primary brain endothelial cells	Cell Biologics	Cat# C57–6023
**Experimental Models: Organisms/Strains**		
Mouse: B6.129S2(D2)-Vtn^tm1Dgi^/J	Dr. Thomas Sisson (Made by Dr. David Ginsburg, Zheng et al., 1995)	RRID:IMSR_JAX:004371
Mouse: *Vtn*^*RGE/RGE*^	Dr. Thomas Sisson (Wheaton et al., 2016)	N/A
Mouse: B6.129-Itga5^tm2Hyn^/J	Jackson Laboratory	RRID:IMSR_JAX:032299
Mouse: B6.129P2(Cg)-Itgav^tm2Hyn^/J	Jackson Laboratory	RRID:IMSR_JAX:032297
Mouse: Cdh5-CreER	Dr. Ralf Adams (Wang et al., 2010)	N/A
**Oligonucleotides**		
siRNA targeting Vitronectin	Thermo Fisher Scientific	Cat# 4457308, siRNA IDs s76001
siRNA targeting Vitronectin	Thermo Fisher Scientific	Cat# 4457308, siRNA ID s76002
Control siRNA	Thermo Fisher Scientific	Cat# 4457289
shRNA targeting integrin α5: CCCAGCAGGGAGTCGTATTTACTCGAGTAAATACGACTCCCTGCTGGG	Sigma-Aldrich	Clone ID TRCN0000262763
shRNA targeting integrin α5: ATCAACTTGGAACCATAATTACTCGAGTAATTATGGTTCCAAGTTGAT	Sigma-Aldrich	Clone ID TRCN0000262922
Scramble shRNA: CCTAAGGTTAAGTCGCCCTCGCTCGAGCGAGGGCGACTTAACCTTAGG	Addgene	Plasmid #1864
**Software and Algorithms**		
Image J	NIH	https://imagej.nih.gov/ij/
ImageJ macro for permeability quantification	Andreone et al., 2017; Chow and Gu, 2017	N/A
Prism 9	GraphPad	https://www.graphpad.com/scientific-software/prism/
ELISA 4PL curve fitting	Arigo Biolaboratories	https://www.arigobio.com/elisa-analysis
**Other**		
*In situ* probe for *Vtn*	ACDBio	Cat# 443601
*In situ* probe for *Pdgfrβ*	ACDBio	Cat# 411381-C3
*In situ* probe for *Pecam1*	ACDBio	Cat# 316721-C2
*In situ* probe for *Itga5*	ACDBio	Cat# 575741
*In situ* probe for *Itgav*	ACDBio	Cat# 513901

## Highlights

1. Pericyte-to-endothelial cell signaling regulates blood-CNS barrier function
2. Pericyte-secreted vitronectin binds to integrin α5 receptor in endothelial cells
3. Activation of integrin by vitronectin inhibits endocytosis in endothelial cells
4. Vitronectin-integrin signaling keeps the barrier tight by suppression of transcytosis
