# Supplementary material for: Pericyte-to-endothelial cell signaling via vitronectin-integrin regulates blood-CNS barrier
Source: Neuron. Author manuscript; Available in PMC 2023 May 18. (PMC9119930; doi:10.1016/j.neuron.2022.02.017)
Supplement: 2 [file NIHMS1784900-supplement-2.pdf]

Supplementary Materials for

Pericyte-to-endothelial cell signaling via vitronectin-integrin regulates blood-CNS barrier

Swathi Ayloo[1, 3], Christopher Gallego Lazo[1], Shenghuan Sun[1, 4], Wei Zhang[2], Bianxiao Cui[2], Chenghua Gu[1*]

[1] Howard Hughes Medical Institute, Department of Neurobiology, Harvard Medical School, Boston, MA 02115, USA

[2] Department of Chemistry, Stanford University, Stanford, California 94305, USA

[3] Current address: Sanofi-GMU, 225 2nd Avenue, Waltham, MA 02451

[4] Current address: Biomedical Sciences Graduate Program, UCSF, San Francisco, CA 94143, USA

[*] Lead Contact: chenghua_gu@hms.harvard.edu

This PDF file includes:

Figures S1-S7

Supplemental Figure Legends

Figure S1. Vitronectin expression in CNS pericytes, Related to Figure 1

(A) Validation of vitronectin antibody (green) in retinas of P7 *Vtn*$^{-/-}$ mice. Vessels (isolectin) in magenta.

(B) In situ hybridization images showing *Vtn* (green), *Pdgfrb* (pericyte gene, magenta) and *Pecam1* (endothelial gene, red) in cortex and cerebellum of P7 mouse.

Figure S2. Leakage seen in retinas of *Vtn*$^{-/-}$ mice is independent of tracer size and leakage persists through adulthood, Related to Figure 2

(A) Co-injection of Sulfo-NHS-Biotin (red) and 10 kDa Dextran (green) shows leakage (arrowheads) of both tracers from vessels (isolectin, blue) in P10 retinas of *Vtn*$^{-/-}$ mice.

(B) Quantification of 10 kDa Dextran leakage in wildtype and *Vtn*$^{-/-}$ mice. n = 5 animals per genotype. Mean ± S.D.; ***$p < 0.001$; Student's t-test.

(C) Leakage assay in 8-week old mice with Sulfo-NHS-Biotin (tracer, green) and vessels (isolectin, magenta). White boxes indicate the region shown in higher magnification images. Tracer-filled neuronal cell bodies (arrowheads) are indicated.

Figure S3. Examples of vessels in cerebellum of *Vtn*$^{-/-}$ mice with basement membranes completely filled with HRP, Related to Figure 4

(C) EM Images of whole cross-sectional view of blood vessels in cerebellum of P10 mice. Lumen is filled with HRP. HRP-filled vesicles (arrowheads), pericytes (P) and nuclei outline (dashed lines) are indicated.

(D) Higher magnification images of boxed regions in (C) shows darker staining of basement membrane (black arrows) on the abluminal side (yellow dashed line) of *Vtn*$^{-/-}$ mice.

(D) Quantification of percentage of vessels with basement membrane filled with HRP in wildtype and *Vtn*$^{-/-}$ mice. n = 4 animals per genotype. Mean ± S.D.; ***$p < 0.001$; Student's t-test.

Figure S4. Lack of vitronectin does not alter vascular basement membrane composition, Related to Figure 5

(A) Tilescan images of collagen IV in P10 retinas of wildtype and *Vtn*$^{-/-}$ mice.

(B) Higher magnification images of collagen IV (green), vessels (isolectin, blue) and pericytes (NG2:DsRed, red).

(C) Representative images of Collagen IV (green) ensheathment of vessels (isolectin, cyan) and pericytes (NG2:DsRed, red) in P10 retinas.

(D, E) Representative western blots (D) and quantification (E) of Fibronectin protein levels normalized to Gapdh in whole retinal lysates of P10 wildtype and *Vtn*$^{-/-}$ mice. n = 3 mice per genotype. Mean ± S.D.; n.s. not significant, $p > 0.05$; Student's t-test.

(F, G) Representative images of perlecan (F) and laminin α4 (G) in green and vessels (isolectin, magenta) in P10 retinas of wildtype and *Vtn*$^{-/-}$ mice. White boxes correspond to regions in the higher magnification images.

(H) Representative images of capillaries ensheathed by astrocyte endfeet. Cross-section of vessel and nucleus highlighted in red dashed lines, pericytes (P) shown in green, astrocyte endfeet (AE) in blue, L indicates vessel lumen filled with DAB.

(I) Quantification of percent of capillary cross-section covered by astrocyte endfeet in cerebellum of P10 wildtype and *Vtn*$^{-/-}$ mice. n = 4 mice per genotype, 15-20 vessels per animal. Mean ± S.D.; n.s. not significant, p > 0.05; Student's t-test.

Figure S5. α5 containing adhesion structures are bonafide focal adhesions, Related to Figure 7

(A, B) Representative images (A) and quantification (B) of α5 adhesion structures in primary brain endothelial cells grown on collagen IV or laminin or vitronectin-coated dishes. n = 15 to 25 cells per condition from 2 independent experiments. Mean ± S.D.; n.s. not significant, p > 0.05, ***p < 0.001; one-way ANOVA with Tukey's post hoc test.

(C) Representative images of α5 containing adhesion structures (green) co-localizing with phospho-FAK (Y397) or paxillin or vinculin (red) in endothelial cells (phalloidin in magenta).

(D) Quantification of percentage of α5 adhesion structures positive for pFAK or paxillin or vinculin. n = 15 to 20 cells per co-staining from 2 independent experiments. In each case, >90% of α5 containing adhesions are positive for these focal adhesion markers.

Figure S6. Endothelial-deletion of *Itga5* does not result in impaired vascular patterning or morphology, Related to Figure 8

(A) Tilescan images showing retinal vasculature of P10 retinas in mice lacking endothelial Itga5.

(B-D) Quantification of vessel density (B), capillary branching (C) and radial outgrowth (D) in retinas of wildtype and *Cdh5:CreER+; Itga5*$^{fl/fl}$ mice. n = 6 animals per genotype. Mean ± S.D.; n.s. not significant, p > 0.05; Student's t-test.

Figure S7. Endothelial RGD-binding integrin, *Itgav*, is not required for CNS barrier integrity, Related to Figure 8

(A) In situ hybridization for *Itgav* (green) *Pecam1* (endothelial gene, red) and *Pdgfrb* (pericyte gene, magenta) in P7 brain tissue. See quantification in Figure 6B.

(B) Sulfo-NHS-Biotin (tracer, green) is confined to vessels (isolectin, magenta) in retinas of P10 *Cdh5:CreER+; Itgav*$^{fl/fl}$ mice. White boxes correspond to higher magnification images in (C).

(C) Tracer (green) confined to vessels with no leakage.

(D) Quantification of vessel permeability in wildtype and *Cdh5:CreER+; Itgav*$^{fl/fl}$ mice. n = 5 animals per genotype. Mean ± S.D.; n.s. not significant, p > 0.05; Student's t-test.

(E) Sulfo-NHS-Biotin (green) is confined to vessels (ICAM2, magenta) in the cerebellum of P10 *Cdh5:CreER+; Itgav*$^{fl/fl}$. White boxes correspond to higher magnification images depicting tracer (green) co-localizing with vessels (ICAM2, magenta).

(F) Quantification of vessel permeability in wildtype and *Cdh5:CreER+; Itgav*$^{fl/fl}$ mice. n = 5 animals per genotype. Mean ± S.D.; n.s. not significant, p > 0.05; Student's t-test.

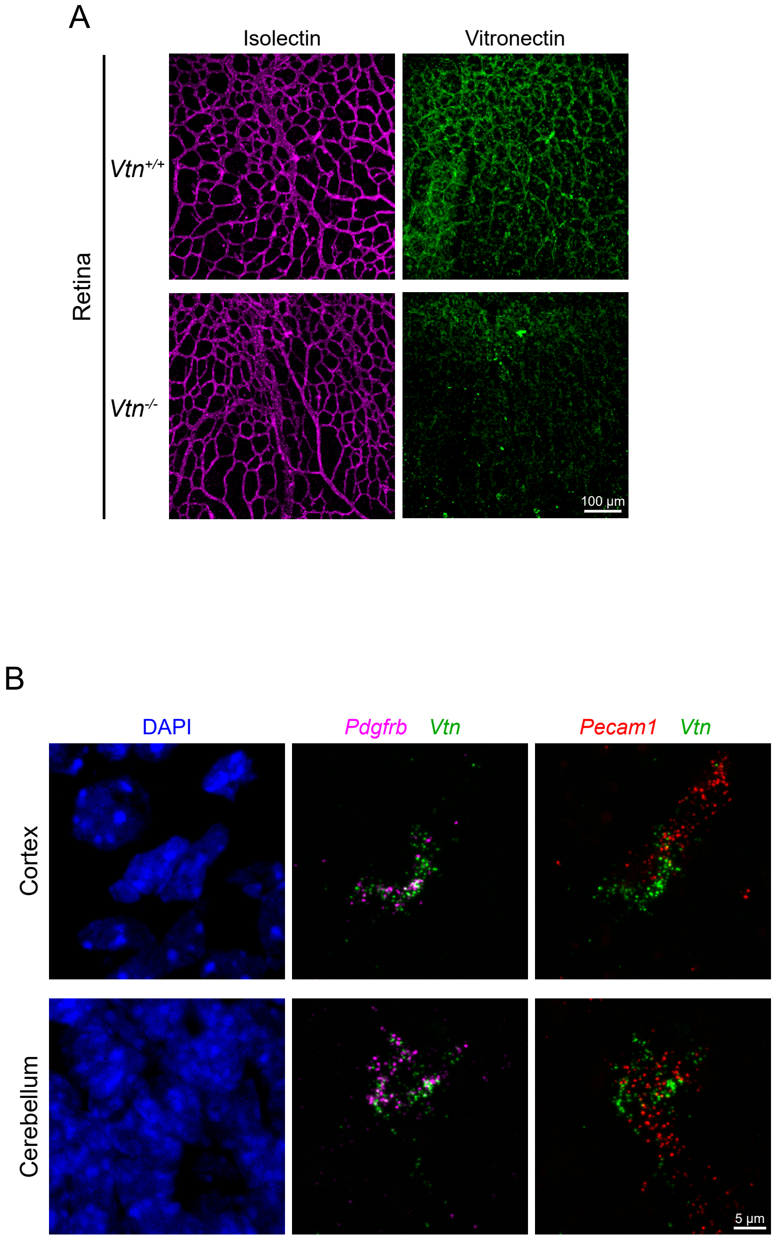

Ayloo et al. Supplementary Figure 1

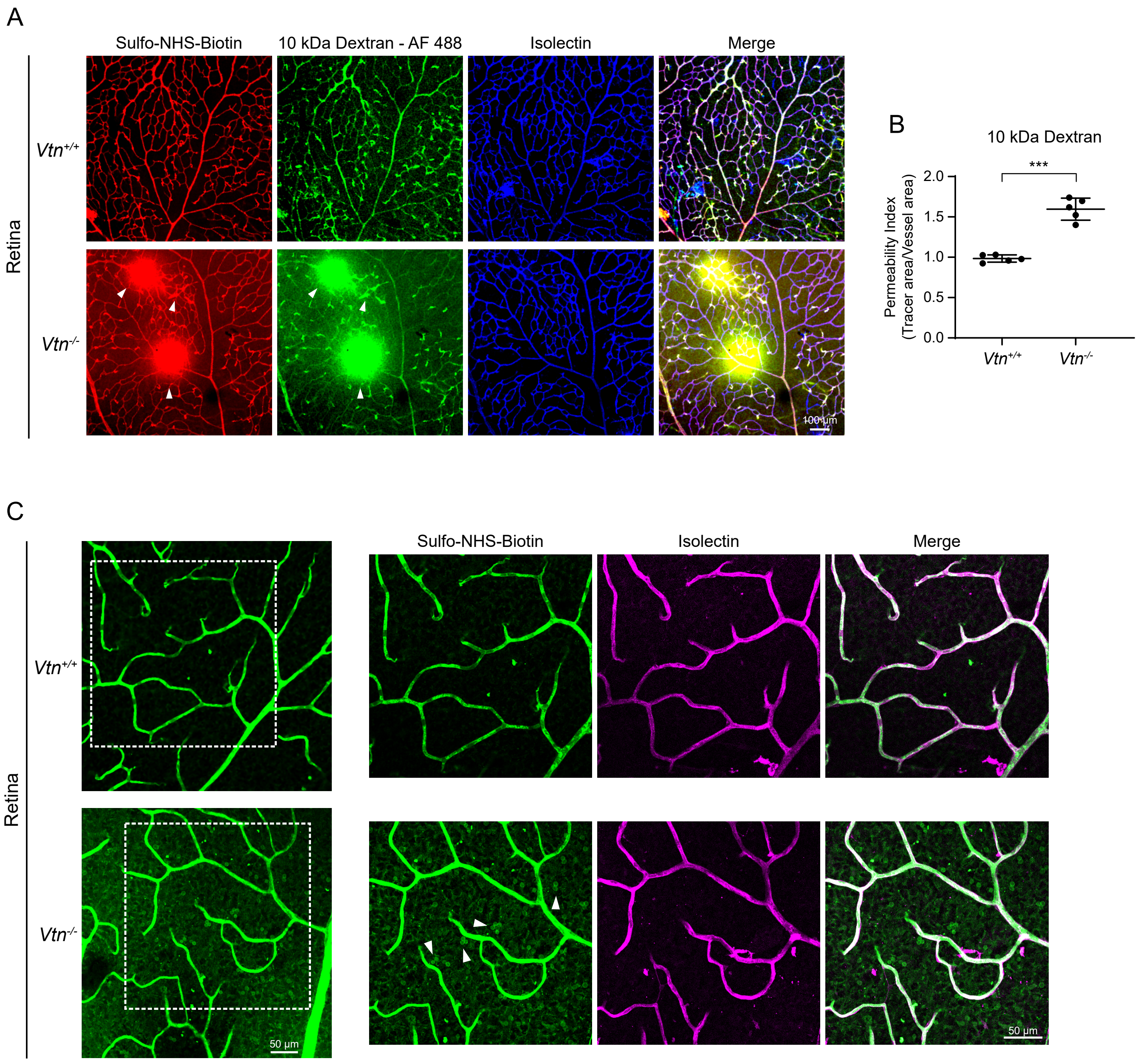

Ayloo et al. Supplementary Figure 2

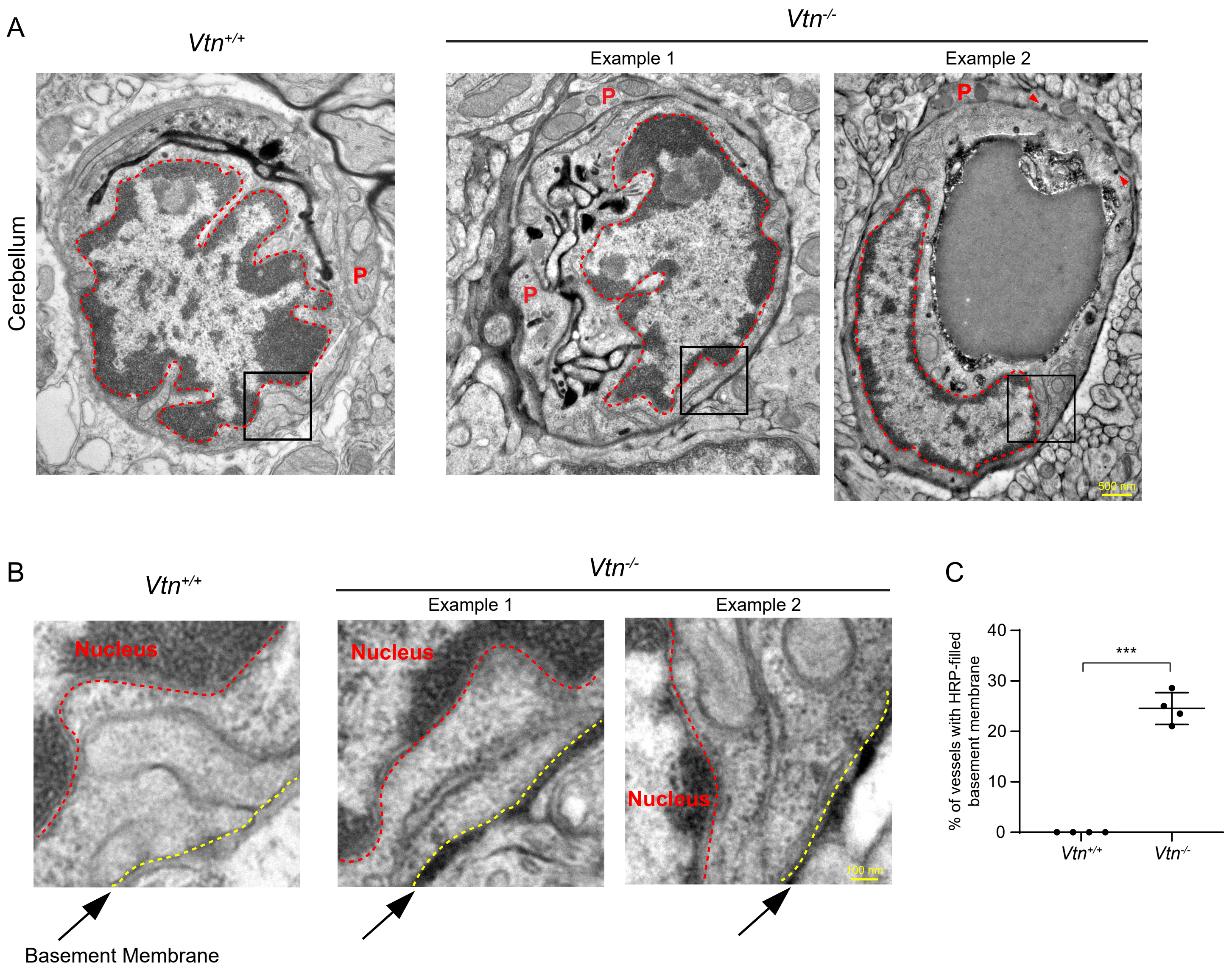

Ayloo et al. Supplementary Figure 3

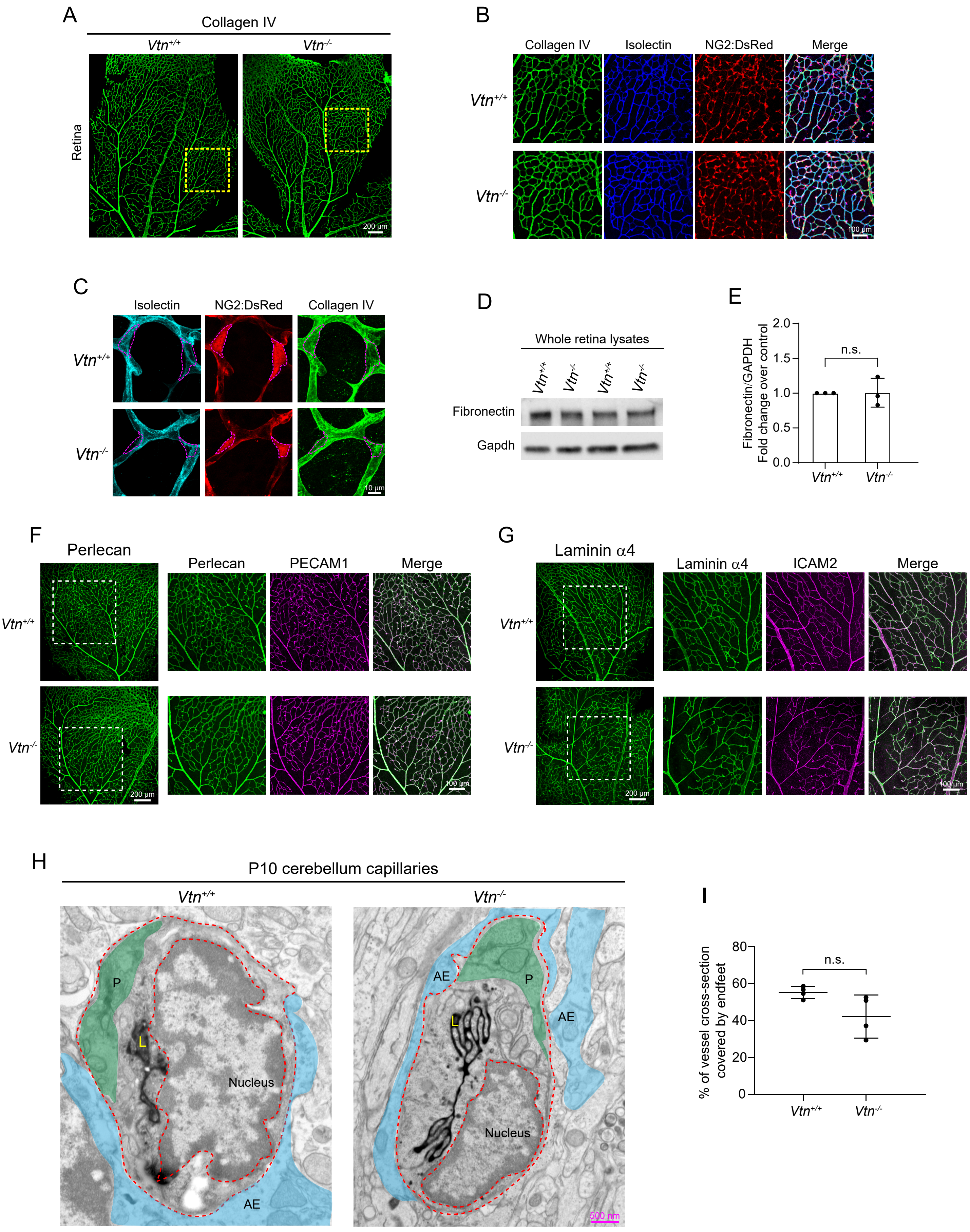

Ayloo et al. Supplementary Figure 4

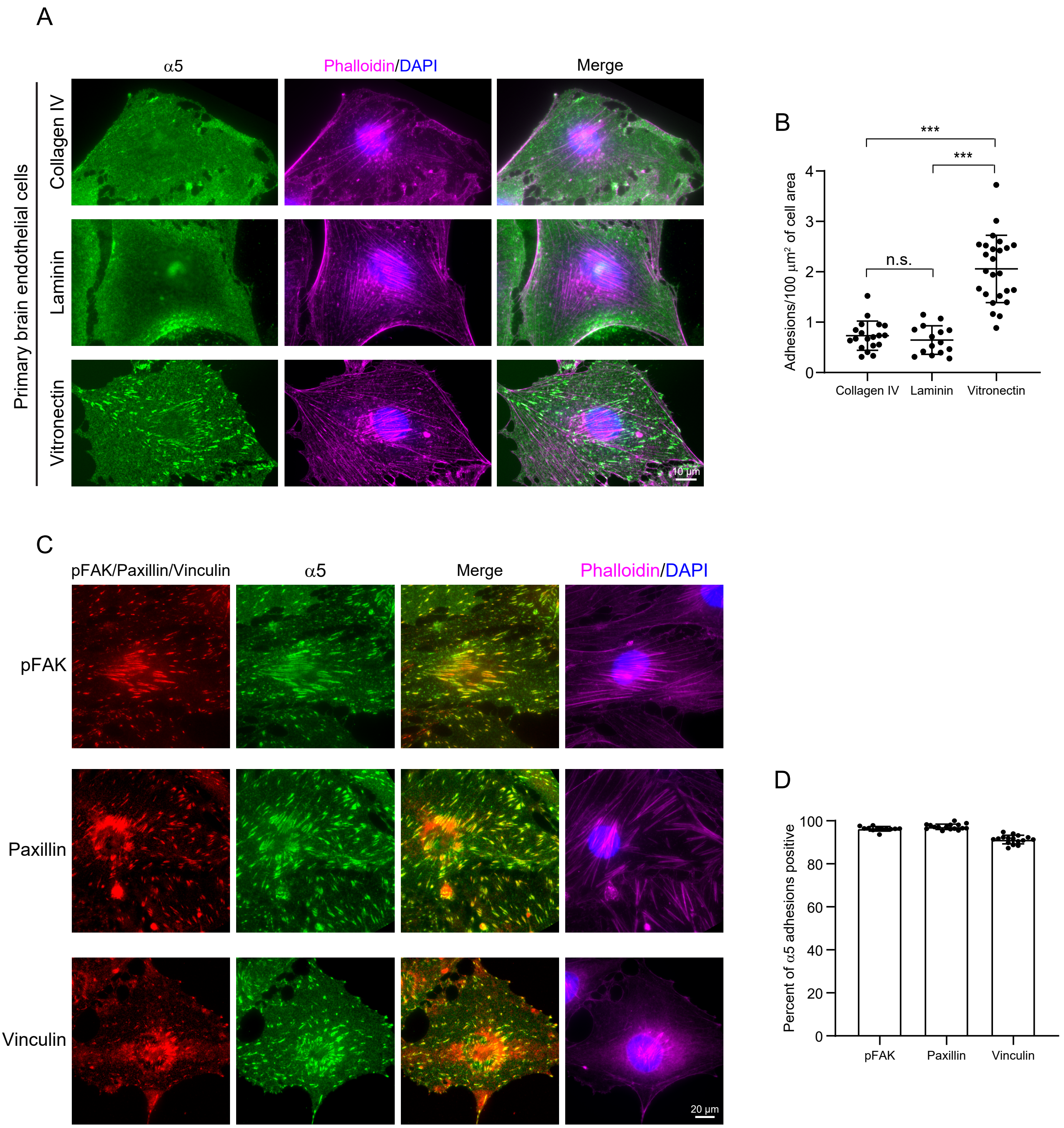

Ayloo et al. Supplementary Figure 5

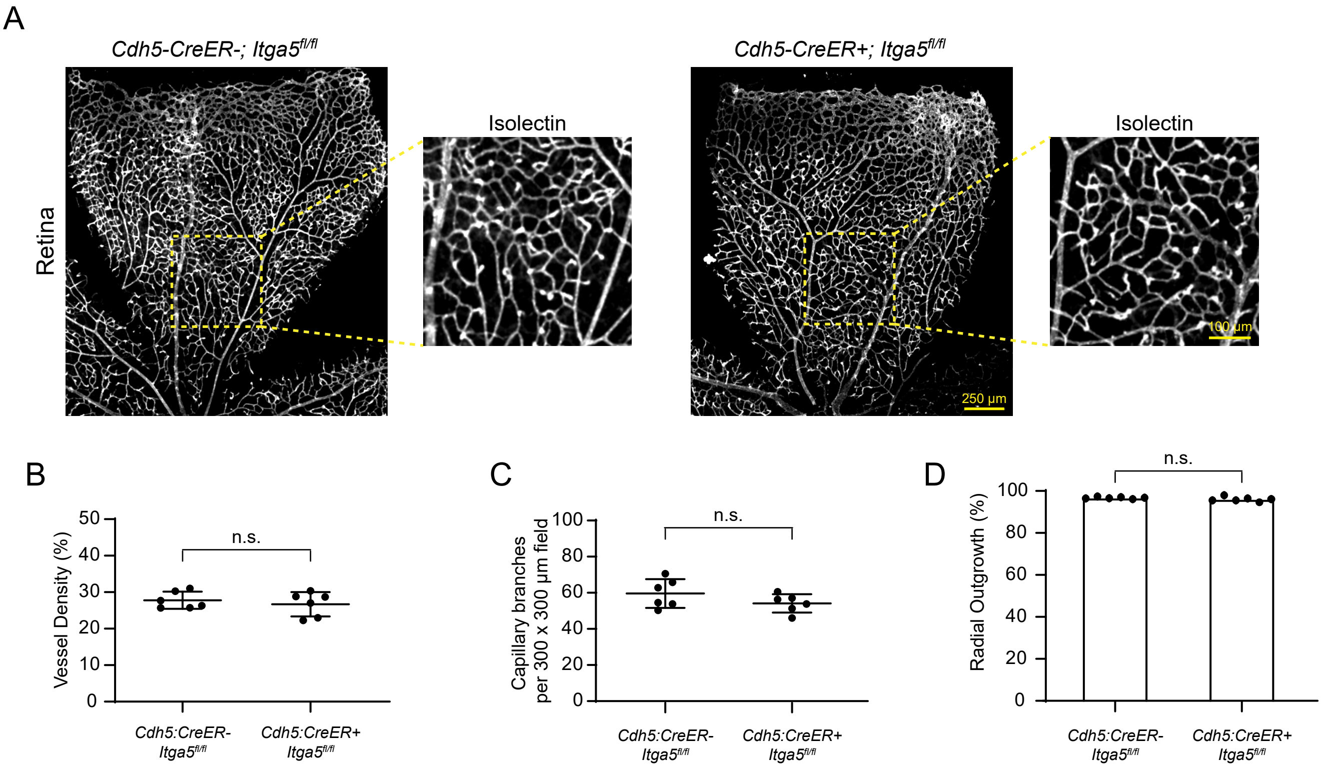

Ayloo et al. Supplementary Figure 6

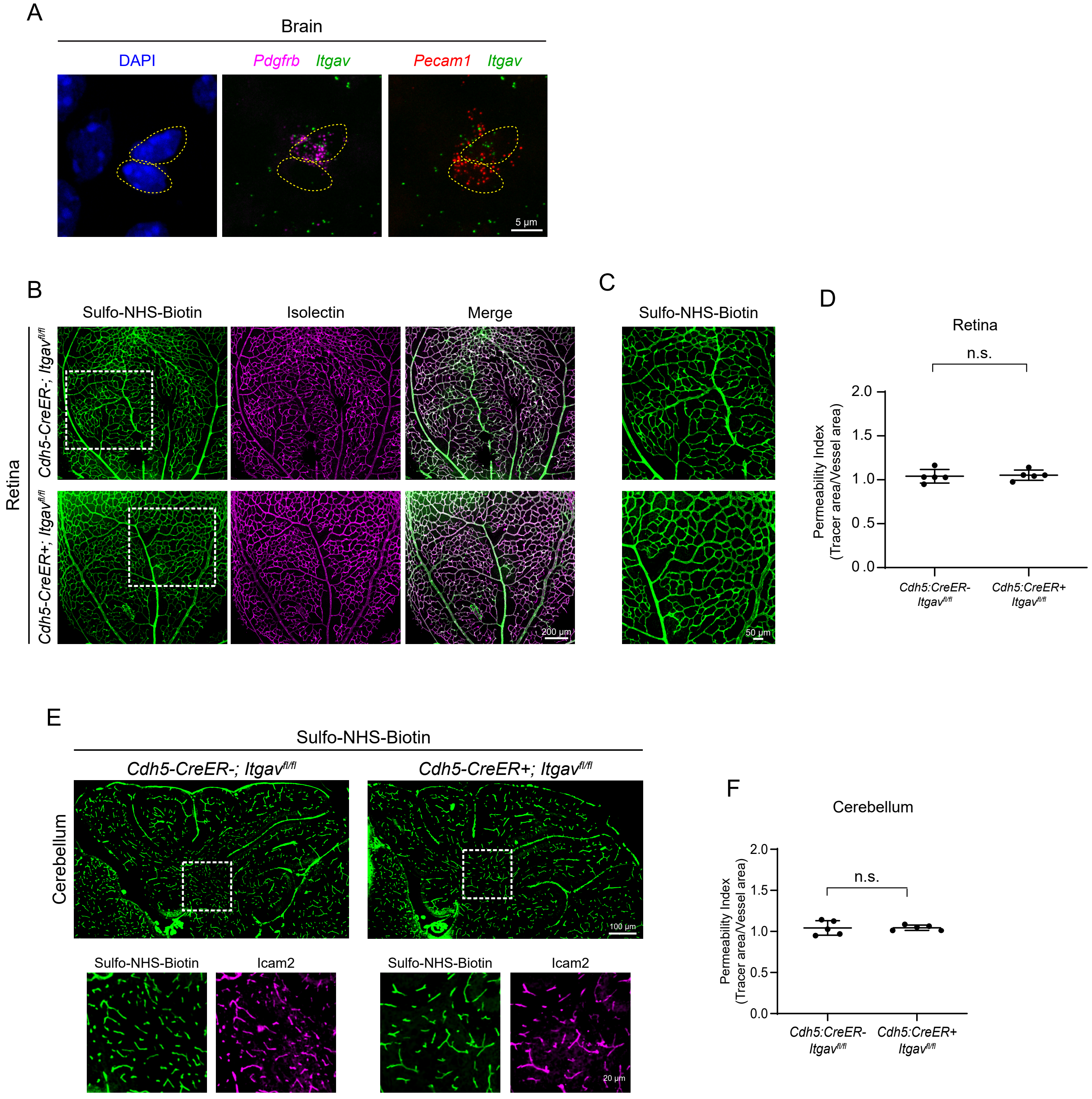

Ayloo et al. Supplementary Figure 7
